# Nocardia rubra cell wall skeleton-induced MARCO expression: implications for improved phagocytosis and cytokine secretion in tumor-associated macrophages

**DOI:** 10.3389/fimmu.2026.1611476

**Published:** 2026-02-12

**Authors:** Guiyuan Zhou, Wei Chen, Xiaomin Dong, Qianyu Guo, Xue Bai, Yan Zheng, Lei Zhang, Suxia Shao

**Affiliations:** 1 Department of Histology and Embryology, Hebei Medical University, Shijiazhuang, Hebei, China; 2 Department of Gynecology, The Fourth Hospital of Hebei Medical University, Shijiazhuang, Hebei, China; 3 Department of Gynecology, The First Hospital of Hebei Medical University, Shijiazhuang, Hebei, China; 4 Shijiazhuang Vocational College of City Economy, Shijiazhuang, Hebei, China

**Keywords:** cervical intraepithelial neoplasia, immune function, macrophage receptors with collagen structure, nocardia rubra cell wall skeleton, tumor-associated macrophages

## Abstract

**Introduction:**

Nocardia rubra cell wall skeleton (Nr-CWS) demonstrates a significant therapeutic effect against human papillomavirus (HPV) infection, but its precise immunomodulatory mechanisms warrant further investigation. This study investigates how Nr-CWS influences tumor-associated macrophages (TAMs) immune functions through macrophage receptor with collagenous structure (MARCO)-mediated mechanisms.

**Methods:**

Cervical tissues of three cervical intraepithelial neoplasia (CIN) patients receiving Nr-CWS monotherapy and HPV infection turning negative were collected before and after treatment, and gene microarray analysis was performed. MARCO expression and immune cell infiltration were further analyzed using transcriptomic data from 33 tumor types in The Cancer Genome Atlas (TCGA). In vitro, TAMs derived from Human Monocytic Leukemia Cell Line 1 (THP-1) cells were treated with Nr-CWS, and changes in MARCO expression, cytoskeletal rearrangement, pseudopod length, lysosome count, and cytokine secretion were assessed. MARCO inhibition experiments were also performed.

**Results:**

Gene microarray revealed significant upregulation of MARCO, a key phagosome pathway gene, post-treatment. TCGA analysis indicated that MARCO expression is significantly altered in most tumor tissues compared to normal tissues and is associated with the infiltration of multiple immune cell types, with a particularly strong correlation to macrophage abundance. Histologically, Nr-CWS increased MARCO⁺ macrophages in cervical tissues. *In vitro*, Nr-CWS elevated MARCO expression in TAMs, enhanced pseudopod formation, lysosome number, cytoskeleton reorganization, and promoted proinflammatory cytokine secretion. Conversely, MARCO inhibition suppressed these immune functions.

**Discussion:**

The study demonstrates that Nr-CWS enhances TAM anti-tumor immune function via MARCO upregulation, leading to improved phagocytic activity and proinflammatory response. These findings align with genomic and cellular evidence, suggesting MARCO as a key mediator in Nr-CWS-induced macrophage reprogramming, with implications for HPV-related neoplasia immunotherapy.

## 1 Introduction

Cervical cancer, which predominantly originates from the cervical transformation zone, represents a highly prevalent malignancy within the female reproductive system. For the development of cervical precancerous lesions and subsequent cancer, the primary causative factor is the persistent infection with high-risk human papillomavirus (HR-HPV) (1, 2). Consequently, the effective management of HR-HPV infection, supported by precise molecular diagnostics (3), is crucial for cervical cancer prevention and treatment. The progression from HPV infection to malignancy depends on both viral persistence and the evasion of host innate and adaptive immunity, which allows the virus to establish a persistent, carcinogenic infection (4). Further improvements in the management of cervical cancer can be expected with the advancement of immunotherapy, which aims to counteract these immune escape mechanisms and enhance the host’s ability to clear HPV-infected and transformed cells (5, 6). Given that the tumor microenvironment, particularly the immune-suppressive milieu, is a critical barrier to effective antiviral and anti-tumor immunity in CIN, agents capable of modulating local immunity have garnered significant interest.

Nocardia rubra cell wall skeleton (Nr-CWS) is recognized as a non-specific immunomodulator with significant clinical efficacy in eradicating human papillomavirus (HPV) infection (7–12). Recent research has demonstrated its ability to enhance immune cell recruitment, activation, maturation, and differentiation within the tumor microenvironment of patients with cervical intraepithelial neoplasia (CIN) (8, 13–15). Furthermore, Nr-CWS has been shown to bolster local immune responses in the cervix, leading to the elimination of HPV infection in cervical tissues and the reversal of low-grade cervical cytological abnormalities. Its proposed mechanism involves the activation of pattern recognition receptors on innate immune cells, initiating a cascade that promotes a more effective anti-viral and anti-neoplastic immune state.

Macrophage receptor with collagenous structure (MARCO), a macrophage receptor in the class A scavenger family (16, 17), plays a dual role in pathogen clearance and reprogramming of tumor-associated macrophages (TAMs) in cancer immune modulation. Its association with immune-related functions of TAMs, including phagocytosis, cytokine secretion, and cellular repolarization, has been established. Research indicates that MARCO plays a significant role in inflammation, facilitating macrophage activation, cytokine production, and contributing to the pathogenesis of various inflammatory conditions (18, 19). Furthermore, MARCO has been shown to facilitate phagocytosis of immune cells, enhance host defense mechanisms against various pathogens, and contribute to crucial cellular processes such as adhesion, migration, and antigen uptake (20, 21). Despite these well-characterized functions in infection and cancer immunology, limited and inconclusive research has been conducted on the specific role of MARCO in the context of HPV infection and the development of cervical precancerous lesions, particularly regarding how Nr-CWS might interact with or influence its expression and function.

In the present study, we first identified a dynamic increase in MARCO expression following Nr-CWS treatment, a finding that stands in contrast to previous reports of elevated MARCO levels in tumor tissues compared to adjacent normal tissues (22)-a static pathological comparison. This highlights the novelty of our study in capturing treatment-induced dynamic changes. And this upregulation was associated with enrichment in several functional categories, such as pattern recognition receptor activity, endocytic vesicle membrane, and cytoskeleton components. And it is closely related to the enrichment of phagosome signaling pathway. We believe this drug-induced upregulation of MARCO may represent a novel immunomodulatory mechanism of Nr-CWS, distinct from its constitutive overexpression in established cancers. Therefore, we focused on MARCO in subsequent investigations. This research examined the regulatory effect of Nr-CWS on MARCO expression in macrophages, both *in vivo* and *in vitro*. Furthermore, it elucidated the function and underlying mechanism of MARCO in the management of HPV infection with Nr-CWS.

## 2 Results

### 2.1 Effect of Nr-CWS against persistent HPV infection

The efficacy of Nr-CWS was assessed in a cohort of 84 recruited patients aged between 21 and 65 years old since 2019. **Table 1** summarizes HPV clearance outcomes stratified by patient age and pathological features. Among the total cohort of 84 patients, the rate of complete HPV clearance was higher in the 21–40 years group (55.9%, 33/59) than in the 41–65 years group (32.0%, 8/25), while the rates of partial clearance were similar between the two groups (8.5% *vs*. 8.0%). The Nr-CWS effective rate was substantially higher in patients aged 21–40 years (64.4%, 38/59) compared to those aged 41–65 years (40.0%, 10/25). When stratified by pathological diagnosis, patients with CIN I (n=56) exhibited a complete clearance rate of 51.8% (29/56), whereas those with CIN II–III (n=28) showed a somewhat lower rate of 42.9% (12/28). The CIN II–III group had a higher rate of partial clearance (14.3%, 4/28) than the CIN I group (5.4%, 3/56). Notably, in contrast to the age-based analysis, the Nr-CWS effective rate was identical between the two pathology groups.

**Table 1:** HPV clearance outcomes stratified by patient characteristics.

Characteristic	Total patients, n	Complete clearance, n (%)	Partial clearance, n (%)	No clearance, n (%)
Age range				
21–40 years	59	33 (55.9)	5 (8.5)	21 (35.6)
41–65 years	25	8 (32.0)	2 (8.0)	15 (60.0)
Pathological features				
CIN I	56	29 (51.8)	3 (5.4)	24 (42.9)
CIN II-III	28	12 (42.9)	4 (14.3)	12 (42.9)

**Figure 1:**
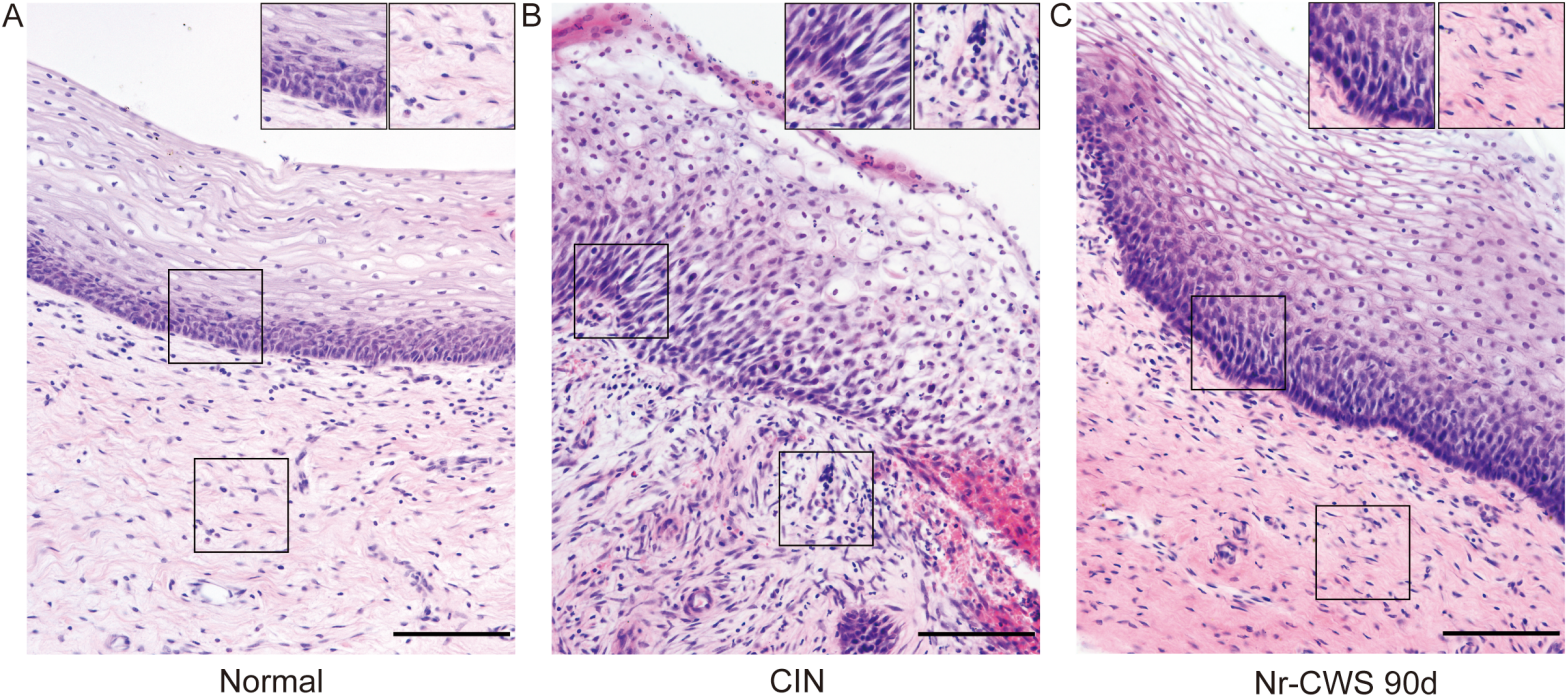
Effect of Nr-CWS on the morphology of cervical tissue structure. The structure of cervical tissues was observed by H&E staining in normal group **(A)**, pre-**(B)** and post-Nr-CWS treatment group **(C)** [**(A–C)** scale bars = 100μm]. The enlarged view in the upper right corner highlights the changes in inflammatory response and basal layer structures in the cervical tissue before and after Nr-CWS treatment.

**Figure 1** illustrates the structural alterations observed in cervical tissues, characterized by enhanced inflammatory cell infiltration and basal layer disruption scores. The cervical tissue predominantly consists of epithelium and lamina propria. In the normal control group, the basal layer cells are arranged neatly, and the cells in the lamina propria are sparsely distributed (**Figure 1A**). In contrast, the CIN group exhibited a notable elevation in cell count and disruption of arrangement in the basal layer, as well as an increased presence of inflammatory cells in the lamina propria (**Figure 1B**). Notably, after 90 days of Nr-CWS treatment, the cervical tissue exhibited a tendency towards normalization (**Figure 1C**).

### 2.2 Gene microarray analysis of Nr-CWS-induced pathways in cervical tissue – highlighting phagocytosis involving MARCO

**Figure 2:**
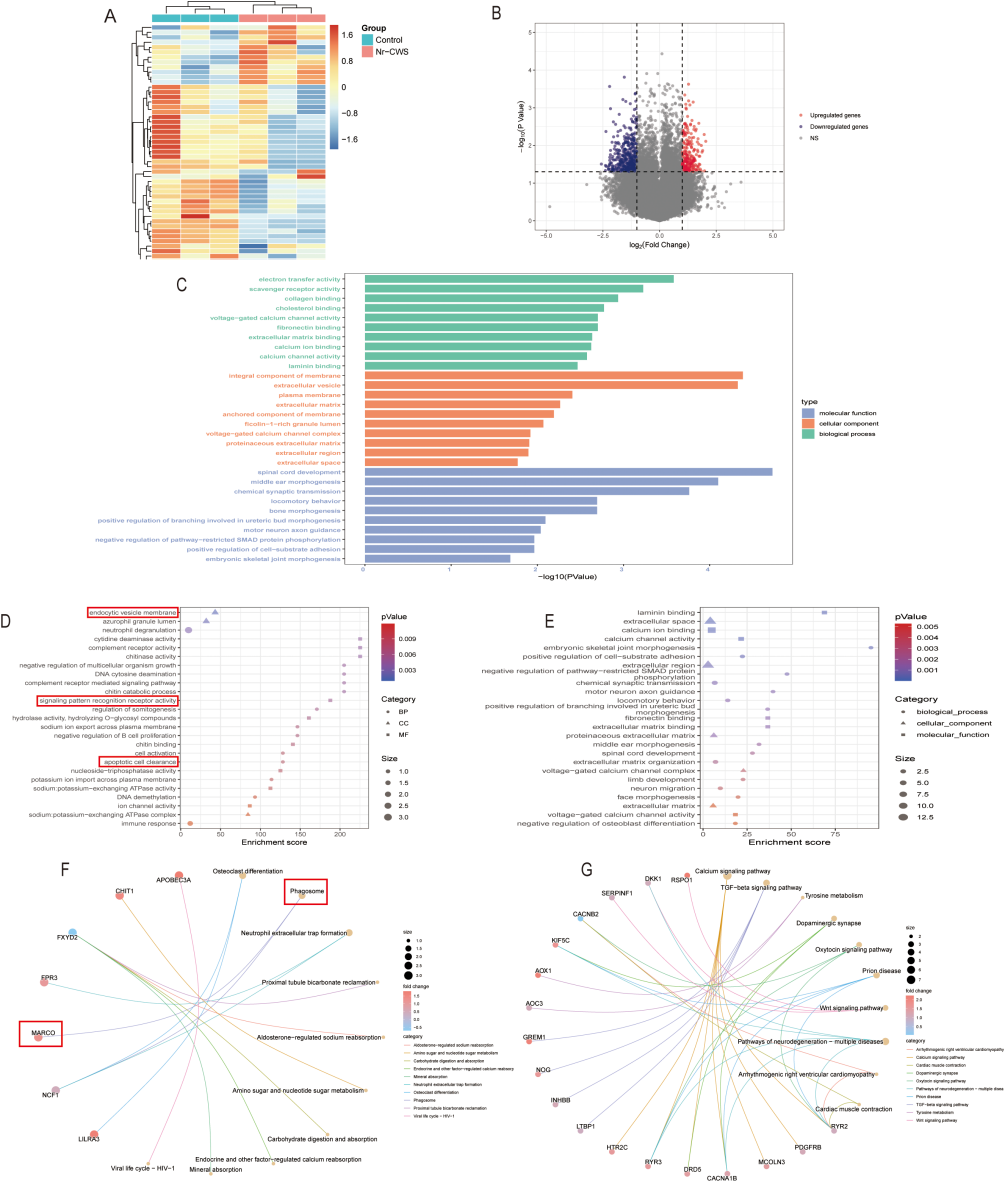
Differentially expressed genes and enriched biological processes in cervical tissues post Nr-CWS treatment. **(A, B)** Heatmap and volcano plot showing the differentially expressed genes between pre- and post-Nr-CWS treatment group. Heatmap **(A)** showed the top 50 genes with significant differences between the Nr-CWS treatment group and the control group. The red color represents the upregulated genes, while the blue color represents the downregulated. Plotted for each gene are the negative log10 of the p-value and the log2 of the fold change of gene expression in volcano plot **(B)**. The colored dots represent genes with a fold change value of −/+ 2 and p value < 0.05, with blue dots representing down-regulated genes and red dots representing up-regulated genes. GO **(C-E)** and KEGG **(F, G)** enrichment analysis showed a close relationship between differential genes and immune-related biological functions (The red box showed the biological processes and signaling pathways enriched by MARCO). n = 3.

Our investigation, utilizing microarray gene expression profiling, identified that Nr-CWS induces a distinct immune-related transcriptional signature in cervical tissues. A heatmap and a volcano plot were presented in **Figures 2A** and **2B**, respectively. Differential analysis revealed that 215 messenger RNAs (mRNAs) exhibited differential expression in cervical tissues of HPV-infected patients following Nr-CWS treatment, compared to pre-treatment levels, including 33 up-regulated and 182 down-regulated genes. Functional enrichment analysis was performed on 33 upregulated mRNAs and 182 downregulated mRNAs. Upon organizing gene functions in ascending order of P-values, it was observed that the differentially expressed genes were mainly enriched in immune-related biological processes (**Figure 2C**). Specifically, the up-regulated genes showed significant enrichment in pathways such as endocytic vesicle membrane, neutrophil degranulation, complement receptor activity, among others (**Figure 2D**), while the down-regulated genes were predominantly associated with laminin binding, calcium ion binding, extracellular space, calcium channel activity, and other related pathways (**Figure 2E**). Notably, as shown in **Figures 2F, G**, we found significant changes in phagosome signaling pathways that may be implicated in immune cell recognition and clearance of pathogens. Among these, particular attention was given to the MARCO gene, which is closely related to multiple biological processes and signaling pathways associated with phagocytosis.

### 2.3 Aberrant expression and prognostic value of MARCO in various cancers

**Figure 3:**
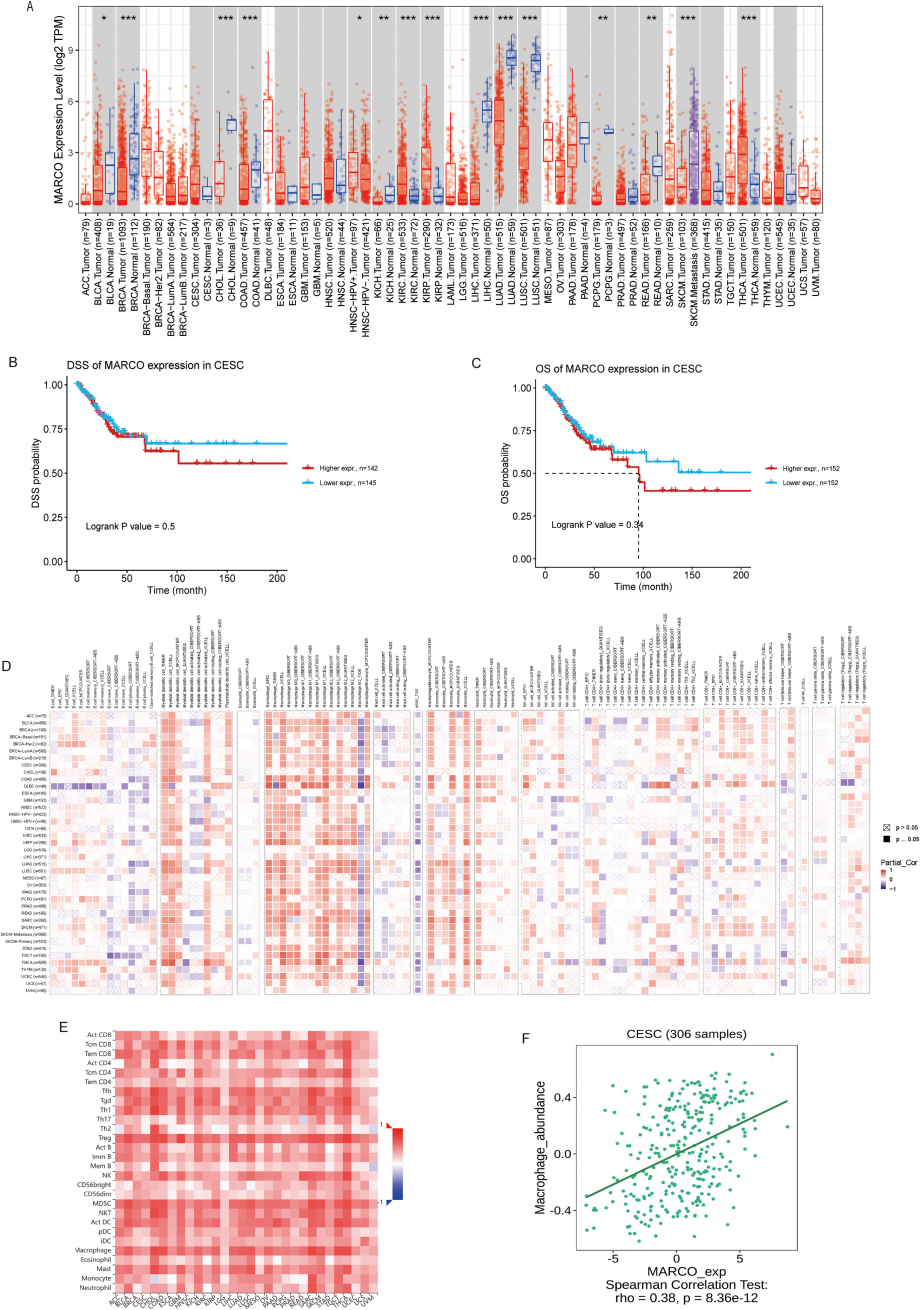
Aberrant expression and prognostic value of MARCO and its relationship with tumor immunity. **(A)** The relative level of MARCO mRNA expression for various cancers in TIMER. **(B, C)** The relationship between MARCO expression and patient prognosis (OS and DSS) in CESC in GSCA. **(D, E)** The associations between MARCO expression and infiltration of various TILs in TIMER and TISIDB. **(F)** The correlations between MARCO and the abundances of macrophage in CESC in TISIDB. ^*^P < 0.05, ^**^P < 0.01, ^***^P < 0.001 *vs*. normal.

First, we evaluated the distribution of MARCO in tumor tissue across all The Cancer Genome Atlas (TCGA) cancers in Tumor Immune Estimation Resource (TIMER) and found that MARCO expression is significantly different compared to normal tissues in the majority of tumors (**Figure 3A**, P < 0.05). Next, Gene Set Cancer Analysis (GSCA) was utilized to examine the association between MARCO and clinical outcome, as depicted in **Supplementary Figures 1–4**, elevated MARCO expression was significantly associated with poorer overall survival (OS) and disease-specific survival (DSS) in kidney renal clear cell carcinoma (KIRC). Conversely, elevated MARCO expression was associated with a more favorable prognosis in skin cutaneous melanoma (SKCM). Although the statistical significance did not reach the conventional threshold, high expression of MARCO in cervical squamous cell carcinoma (CESC) showed a trend toward association with poorer prognosis (**Figures 3B, C**). Our previous research has demonstrated a significant association between MARCO and the functions of various immune cells (23). Based on integrated data from the TIMER and GSCA databases, we propose that MARCO may modulate diverse immune responses, potentially resulting in varying tumor outcomes.

### 2.4 Association of MARCO with the tumor immune microenvironment

To further validate MARCO’s role in the immune response, we probed the connection between MARCO expression and immune infiltration. TIMER (**Figure 3D**) and An Integrated Repository Portal for Tumor-immune System Interactions (TISIDB)(**Figure 3E**) were used to investigate the link between MARCO expression and immune cells in a variety of malignancies. The results revealed an association between MARCO expression across various tumors and immune cell infiltration, notably demonstrating a particularly strong association with macrophages. For example, **Figure 3F** and **Supplementary Figures 5–9** show that MARCO expression was positively correlated with the infiltration of macrophages in cancers including bladder urothelial carcinoma (BLCA), cervical squamous cell carcinoma and endocervical adenocarcinoma (CESC), cholangiocarcinoma (CHOL), etc. Integrating the data from the two databases and previous studies, we conclude that MARCO expression in tumor tissues is closely associated with macrophage-related immune response.

### 2.5 Nr-CWS treatment resulted in an upregulation of MARCO expression in cervical tissues

**Figure 4:**
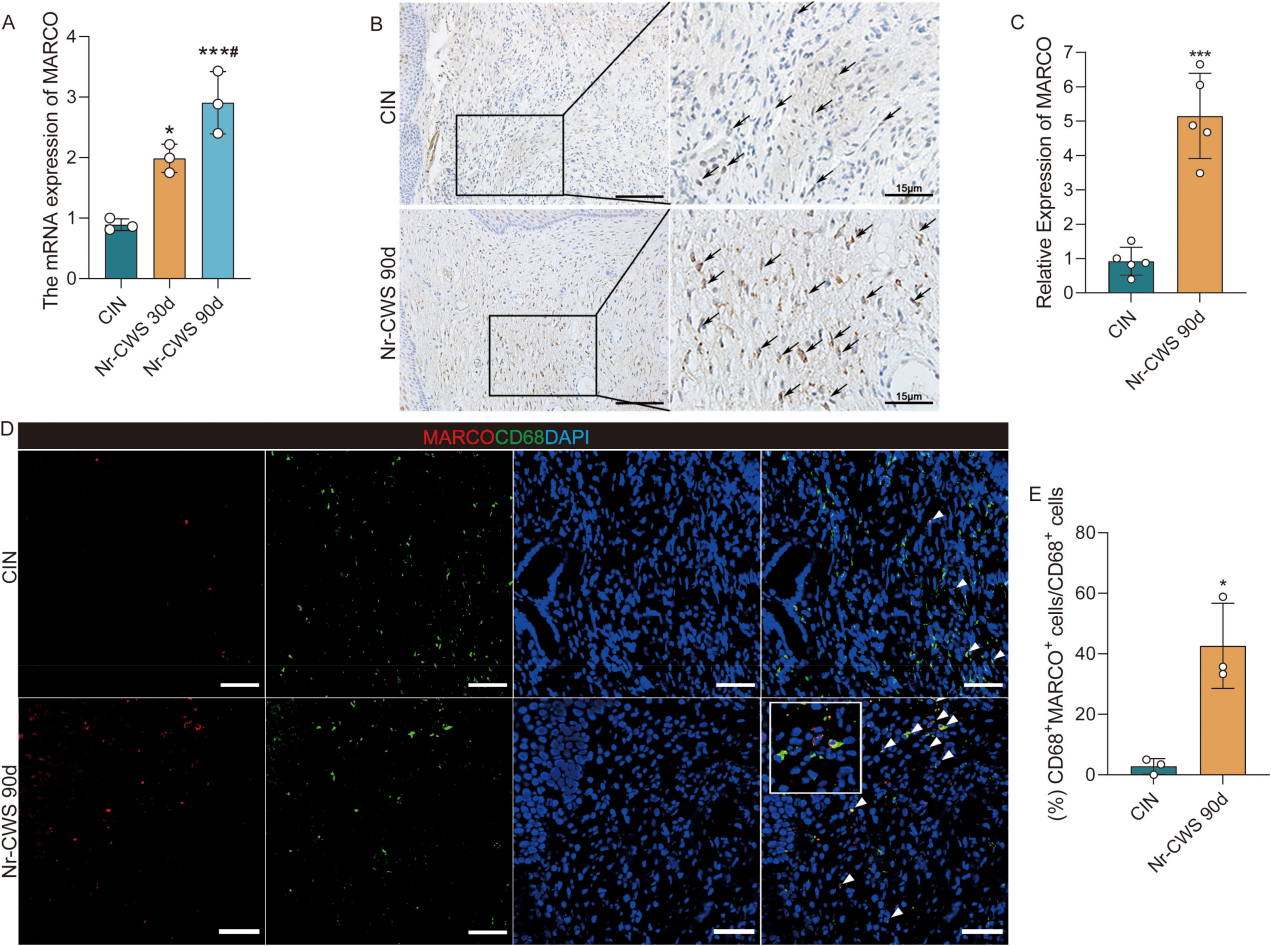
Nr-CWS upregulates MARCO expression in cervical tissues. The expression levels of MARCO were detected by RT-qPCR [**(A)**, n = 3] and immunohistochemistry [**(B, C)**, n = 5, scale bars = 100μm]. The co-expression of CD68 and MARCO was detected by double-labeling immunofluorescence histochemistry pre-and post-Nr-CWS treatment [**(D, E)**, n = 3, scale bars = 50μm]. ^*^P < 0.05, ^**^P < 0.01, ^***^P < 0.001 *vs*. CIN, ^#^*P* < 0.05 *vs*. Nr-CWS 30d.

To verify whether the MARCO expression in cervical tissue changes after Nr-CWS treatment, Reverse Transcription Quantitative Polymerase Chain Reaction (RT-qPCR) analysis was conducted on cervical tissue samples pre- and post-treatment to quantify MARCO mRNA levels. A notable upregulation of MARCO expression was observed after 30 days of Nr-CWS treatment compared to the CIN group, with a more pronounced increase after 90 days (**Figure 4A**, P < 0.05). Immunohistochemical staining was conducted on cervical tissue samples pre- and post-treatment to verify the protein expression levels of MARCO. Consistent results were observed in immunohistochemical staining, indicating a significant increase in both the number and intensity of MARCO-positive cells in cervical tissue following Nr-CWS treatment (**Figures 4B, C**, P < 0.05). These results suggest a heightened MARCO expression in patients treated with Nr-CWS. Our previous H&E staining results demonstrated that Nr-CWS treatment alters inflammatory cell infiltration. This prompted a key question: which specific immune population is linked to the observed changes in MARCO expression? Guided by our prior bioinformatic analysis and supported by CD68 immunohistochemical staining of cervical tissues before Nr-CWS treatment (**Supplementary Figure 10**), which collectively pointed to tumor-associated macrophages (TAMs), we next sought to experimentally validate the correlation between Nr-CWS-induced MARCO upregulation and this specific cell type. We performed an analysis of cell subsets expressing MARCO in patients through dual-color immunofluorescence. CD68, a well-established macrophage marker, was utilized in this analysis. The results displayed in **Figures 4D** and **E** revealed a significant increase in CD68^+^MARCO^+^/CD68^+^ cell ratio, indicating that MARCO upregulation occurred predominantly in macrophages following Nr-CWS treatment. These findings demonstrate a strong association between MARCO expression and cervical tissue macrophages, suggesting that the up-regulation of MARCO in these macrophages potentially may play a crucial role in mediating the effect of Nr-CWS on macrophage-driven anti-tumor immunity.

### 2.6 Nr-CWS promotes MARCO expression in TAMs

**Figure 5:**
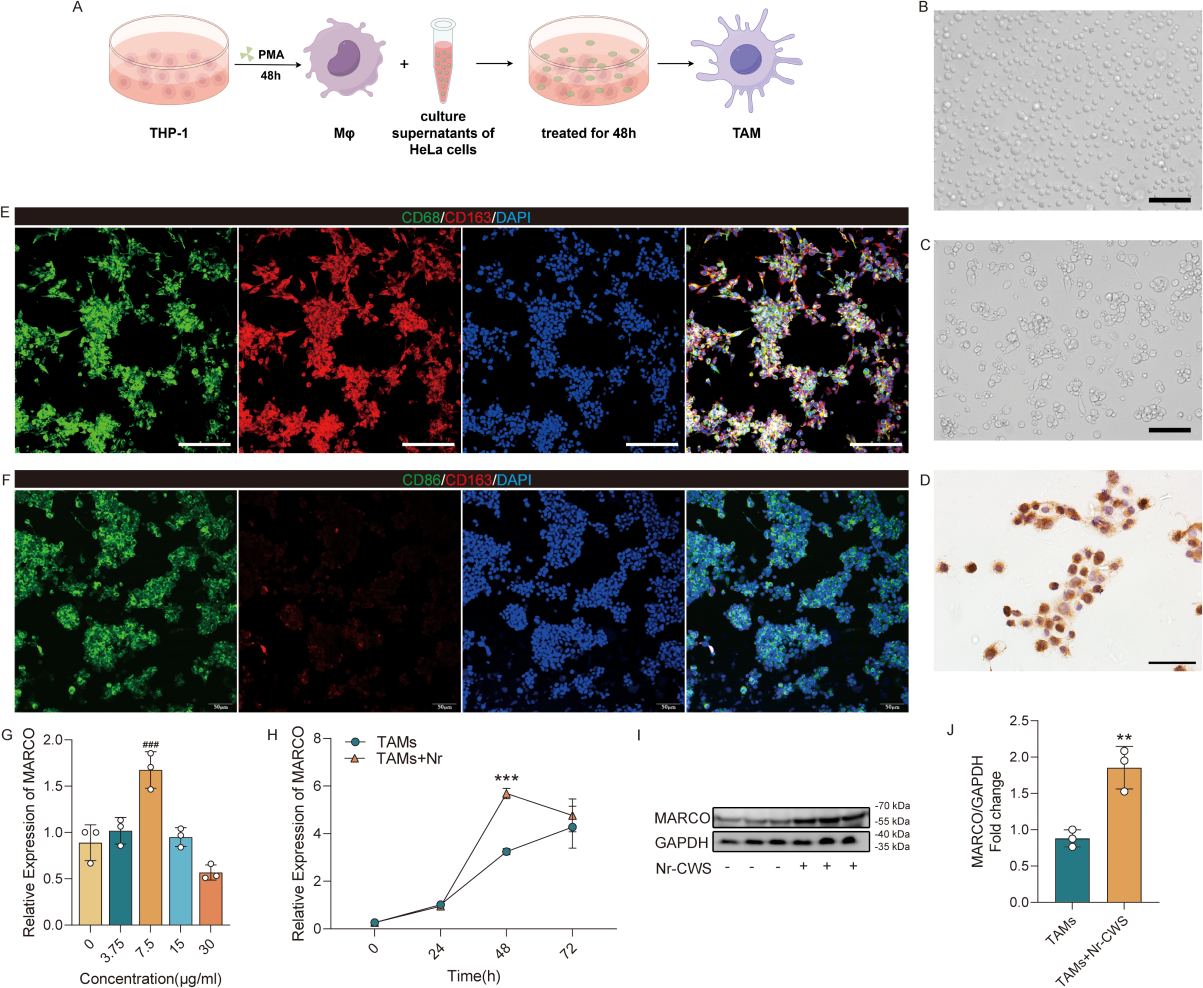
Nr-CWS promotes MARCO expression in TAMs. THP-1 cells were first differentiated into macrophages, and then treated with the culture supernatants of HeLa cells for 48 h to obtain TAMs **(A)**. The cell morphology changed during the differentiation of THP-1 cells **(B)** to macrophages **(C)**, and the induced cells were identified by ICC **(D)**. Dual-label immunofluorescence staining showing the phenotypic shift in TAMs induced by Nr-CWS. **(E)** Pre-treatment TAMs are primarily M2-polarized. **(F)** Post-treatment TAMs switch to an M1-polarized state. Optimal Nr-CWS concentration **(G)** and time **(H)** for MARCO expression in TAMs was determined by RT-qPCR. Nr-CWS promotes MARCO protein expression at 7.5μg/mL, 48h treatment detected by WB **(I, J)**. [**(B, C, E, F)**, scale bars = 100μm; D, scale bars = 50μm]. n = 3, ^**^P < 0.01, ^***^P < 0.001 *vs*. TAMs, ^###^*P* < 0.001 *vs*. 0μg/mL.

To determine MARCO expression in macrophages during Nr-CWS treatment of HPV infection, a TAMs model was established for further *in vitro* investigations, as depicted in **Figure 5A**. Human Monocytic Leukemia Cell Line 1 (THP-1) cells were induced to macrophages through incubation with phorbol 12-myristate 13-acetate (PMA), followed by further differentiation into TAMs through exposure to culture supernatants from HeLa cells. The non-adherent THP-1 cells became adherent after treatment with PMA (**Figures 5B, C**), and exhibited CD68 positivity (**Figure 5D**). Subsequent exposure to HeLa cell culture supernatants successfully induced the differentiation of TAMs, as indicated by the expression of the M2 marker CD163 (**Figure 5E**). Conversely, following Nr-CWS treatment, these TAMs underwent repolarization from the M2 to the M1 phenotype (**Figure 5F**).

RT-qPCR and Western blotting (WB) analysis were conducted on TAMs both before and after Nr-CWS administration to assess the mRNA and protein levels of MARCO. RT-qPCR analysis revealed that treatment with 7.5 μg/mL Nr-CWS for 48 hours yielded the highest MARCO expression level, establishing these as the optimal experimental parameters (**Figures 5G, H**). These specific parameters were subsequently utilized in further experiments. Consistent results were observed in Western blot analysis, where treatment with Nr-CWS significantly upregulated MARCO expression (**Figures 5I, J**, P < 0.05). Furthermore, immunofluorescence double staining revealed enhanced expression and co-localization of MARCO with the M1 marker CD86 post-treatment (**Supplementary Figure 11**), reinforcing the specific link between MARCO and the M1 macrophage phenotype.

### 2.7 Nr-CWS enhance the phagocytosis of TAMs

**Figure 6:**
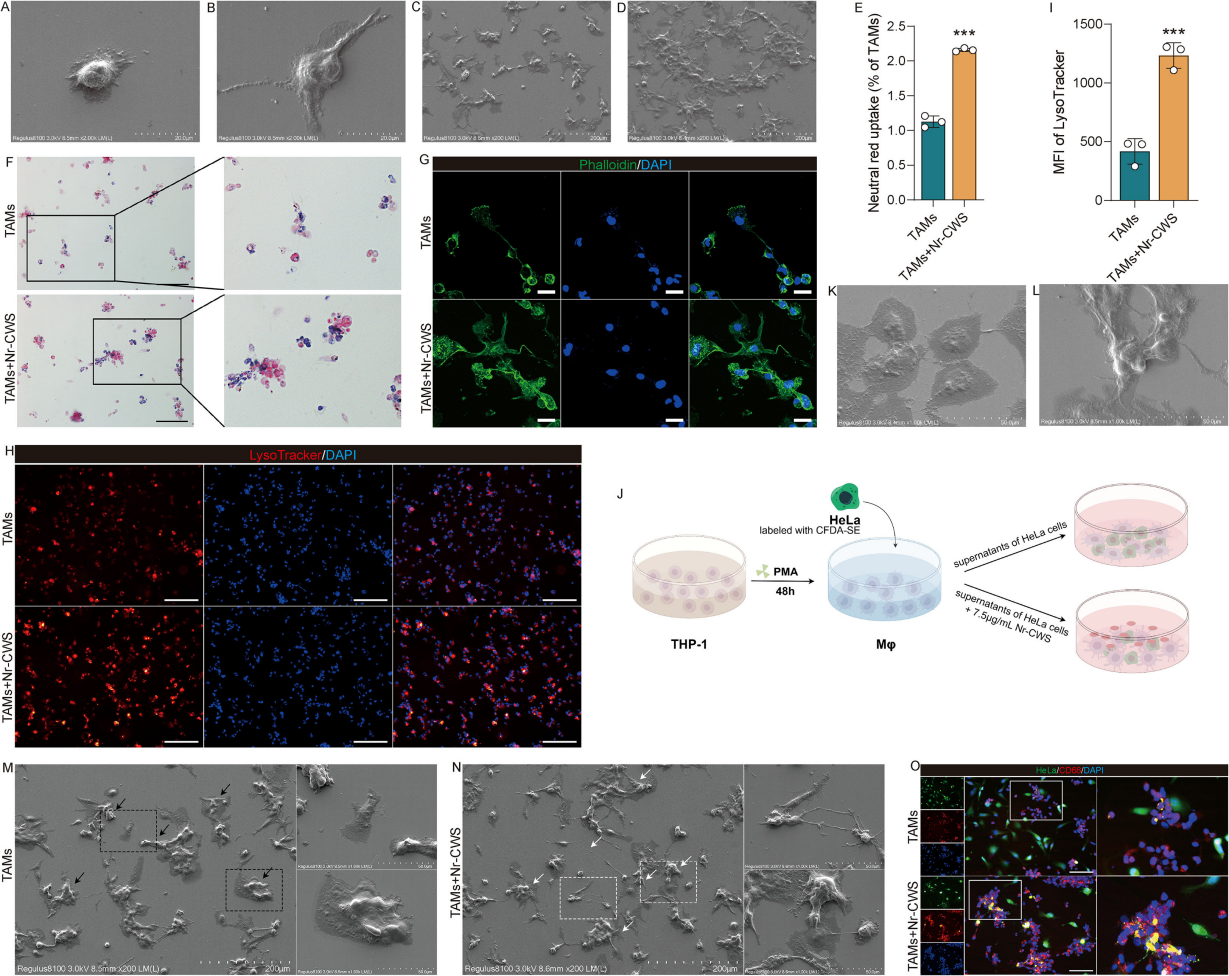
Nr-CWS enhance the phagocytosis of TAMs. The morphology of TAMs changed before **(A, C)** and after **(B, D)** Nr-CWS treatment [**(A, B)**, scale bars = 20μm; **(C, D)**, scale bars = 200μm). Neutral red **(E)** and trypan blue [**(F)**, scale bars = 100μm] uptake assays confirmed the increased phagocytic activity of TAMs. Additionally, Nr-CWS treatment led to rearrangement of the TAM cytoskeleton [**(G)**, scale bars = 30μm] and increased acidification of phagosomes [**(H, I)**, scale bars = 100μm]. **(J-O)** The co-culture system of HeLa and TAMs. HeLa cells were co-cultured with TAMs cells **(J)**, noticeable alterations in cell morphology [**(M, N)**, scale bars = 200μm] as observed through SEM, in comparison to HeLa [**(K)**, scale bars = 50μm] and TAMs [**(L)**, scale bars = 50μm] cultured individually. Additionally, fluorescence staining revealed that Nr-CWS promoted the phagocytosis of HeLa cell debris by TAMs [**(O)**, scale bars = 100μm]. n = 3, ^***^P < 0.001 *vs*. TAMs.

To investigate the effect of Nr-CWS on TAMs function, we first examined morphological changes in TAMs after treatment. Scanning electron microscopy (SEM) revealed an increase in pseudopod length (**Figures 6A, B**) and the formation of larger cell clusters (**Figures 6C, D**) post-Nr-CWS treatment. Based on these morphological changes, we hypothesized that Nr-CWS may enhance the macrophages phagocytic activity. To test this, we performed neutral red and trypan blue uptake assays, which revealed a significant increase in both the neutral red uptake rate (**Figure 6E**, P < 0.05) and the number of internalized trypan blue particles (**Figure 6F**) in the TAMs + Nr-CWS group. The process of macrophage phagocytosis serves as the primary defense mechanism against pathogens, with cytoskeleton rearrangement and phagosome acidification being crucial steps for macrophages in pathogen clearance. To evaluate whether Nr-CWS influences these processes, we utilized phalloidin to mark the cytoskeleton protein and the pH-sensitive dye LysoTracker to assess the number of lysosomes. Our findings indicate that the expression of filamentous actin (F-actin) and the number of lysosomes were all enhanced in the TAMs + Nr-CWS group following Nr-CWS administration, as compared to levels prior to treatment (**Figures 6G–I**, P < 0.05).

Subsequently, TAMs were co-cultured with HeLa cells, as illustrated in **Figure 6J**. Scanning electron microscopy revealed that HeLa cells exhibited a polygonal shape with fine microvilli on the surface (**Figure 6K**), whereas TAMs displayed an irregular shape with prominent bumps on the cell surface (**Figure 6L**). The morphology of HeLa cells appeared normal prior to Nr-CWS treatment, with TAMs and HeLa cells exhibiting relatively independent growth patterns in the co-culture model of TAMs and HeLa cells (**Figure 6M**, indicated by the black arrow). However, after Nr-CWS treatment, HeLa cells showed clear morphological changes, including a decrease in cell volume and the presence of small spherical protrusions on the cell surface-classic hallmarks of apoptotic and necrotic cell death. Additionally, attachment of TAMs to HeLa cells was evident, as indicated by white arrows in **Figure 6N**. Furthermore, fluorescence microscopy confirmed a pronounced increase in HeLa-derived cellular debris (**Figure 6O**, **Supplementary Figure 12**). This debris accumulation signifies heightened cytolysis of HeLa cells, while its concurrent clearance underscores a significantly augmented phagocytic activity by TAMs. These findings indicate that Nr-CWS modulates the phagocytic activity of TAMs, leading to enhanced killing and phagocytosis of HeLa cells by TAMs.

### 2.8 Nr-CWS modulates cytokine secretion in tumor-associated macrophages

**Figure 7:**
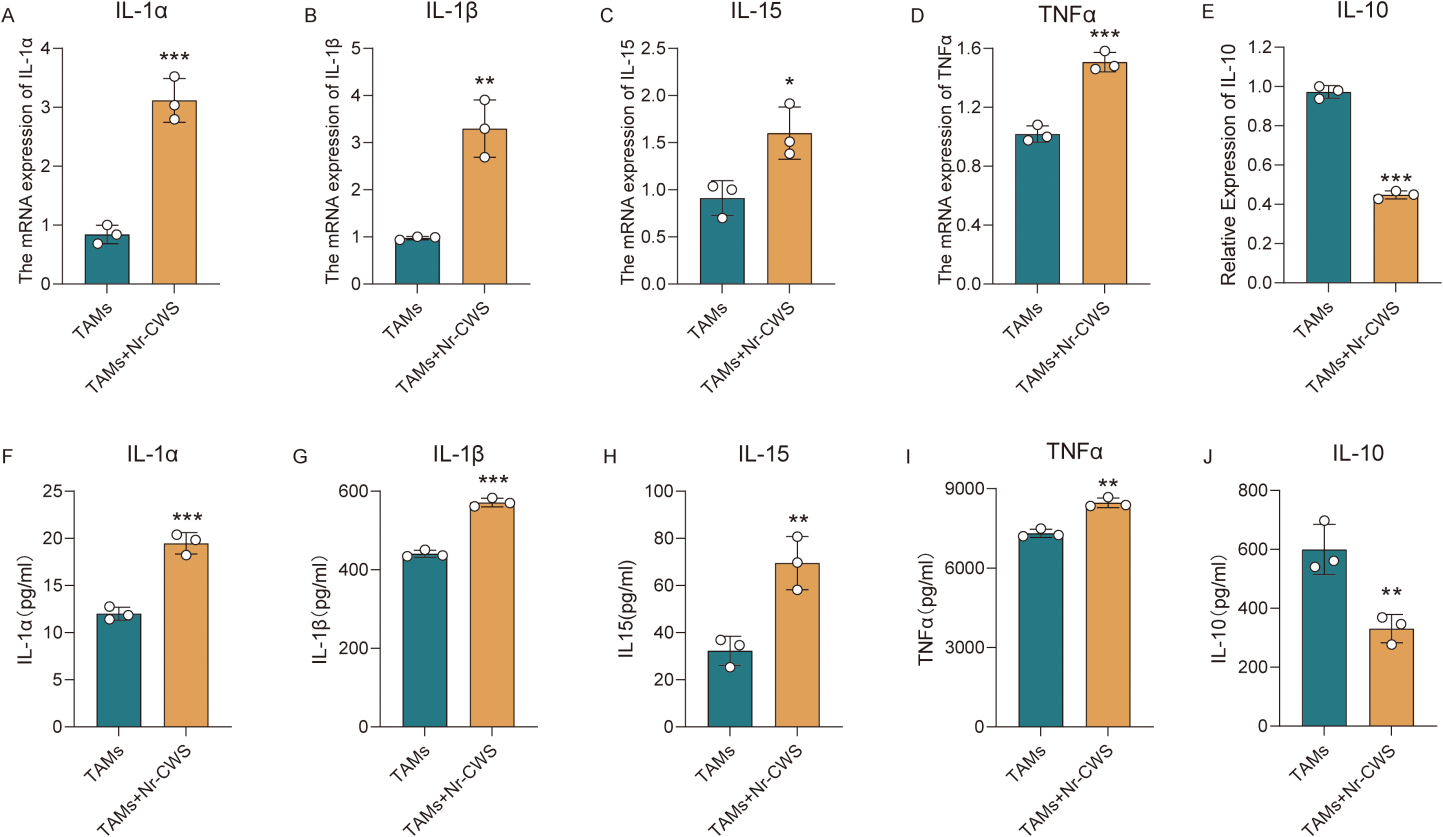
Nr-CWS Modulates Cytokine Secretion in Tumor-Associated Macrophages. The mRNA expression levels **(A-E)** and protein secretion levels **(E-J)** of IL-1α, IL-1β, IL-15, TNFα and IL-10 in TAMs were significantly increased following Nr-CWS administration. n = 3, ^*^P < 0.05, ^**^P < 0.01, ^***^P < 0.001 *vs*. TAMs.

TAMs secrete a diverse array of cytokines that remodel the tumor microenvironment, modulate the activity of other immune cells, and directly impact tumor cells. To assess the impact of Nr-CWS on TAMs cytokine secretion function, we utilized RT-qPCR (**Figures 7A–E**) and Enzyme-Linked Immunosorbent Assay (ELISA) (**Figures 7F–J**) techniques to measure the expression levels of five cytokines, namely interleukin-1 alpha (IL-1α), interleukin-1 beta (IL-1β), interleukin-15 (IL-15), tumor necrosis factor alpha (TNFα), and interleukin-10 (IL-10). Our results show a significant increase in the expression of four pro-inflammatory cytokines (**Figures 7A–D, F–I**), in contrast to a decrease in the anti-inflammatory cytokine IL-10 (**Figures 7E, J**), in the TAMs + Nr-CWS group (all *P* < 0.05). These findings indicated that Nr-CWS could reprogram TAMs into a pro-inflammatory phenotype.

### 2.9 Nr-CWS promotes TAMs phagocytosis and secretion activity via MARCO *in vitro*

**Figure 8:**
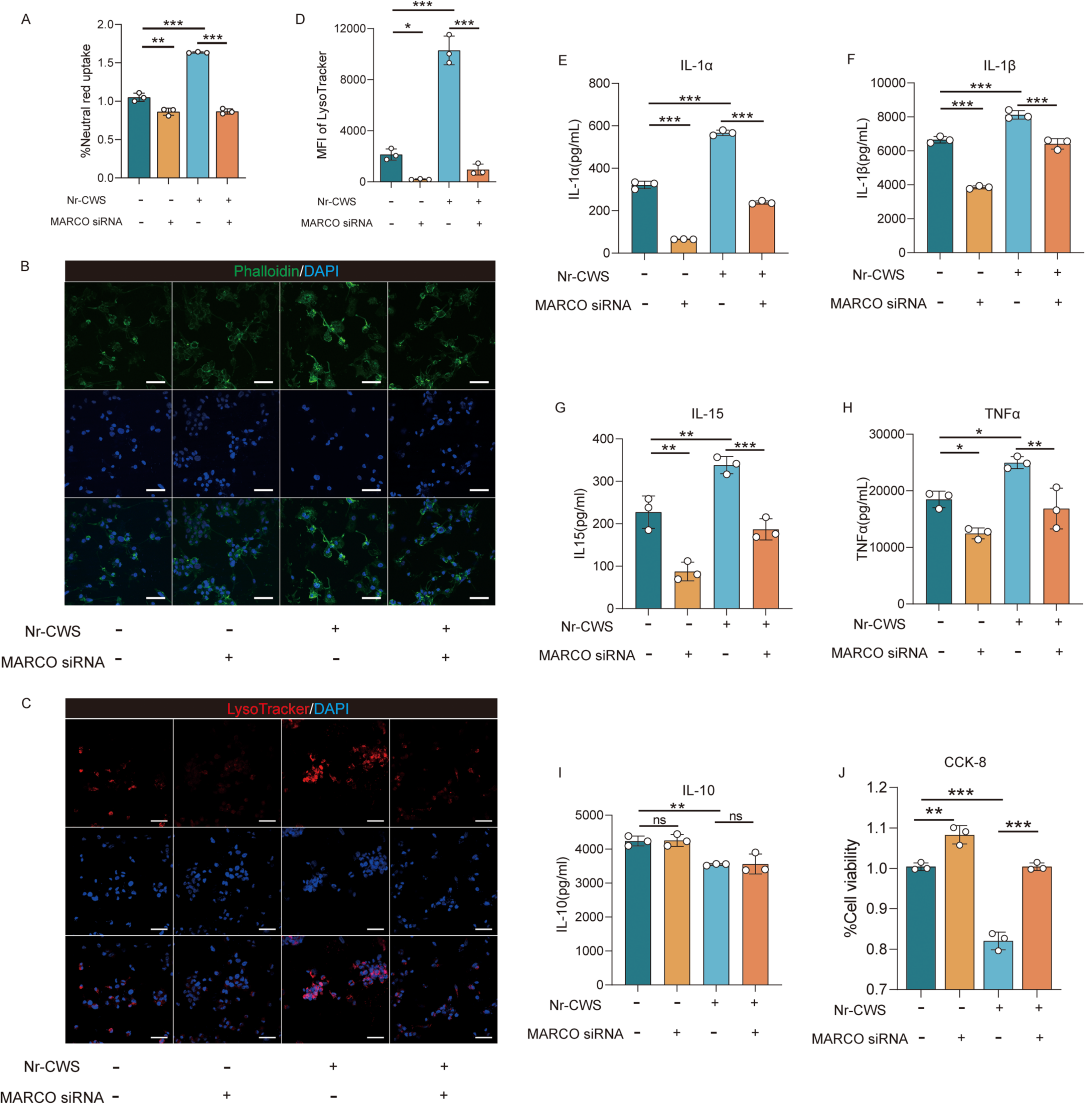
The enhancement of TAMs immune function by Nr-CWS is dependent on MARCO. Results from neutral red uptake experiments demonstrated a significant decrease in phagocytic capacity of TAMs following MARCO knockdown **(A)**. MARCO plays a crucial role in regulating distribution of cytoskeleton in TAMs as well as phagosome acidification, as evidenced by the observed reduction in cytoskeletal rearrangement [**(B)**, scale bars = 100μm] and phagosome acidification [**(C**, **D)**, scale bars = 100μm] upon MARCO expression suppression. Furthermore, following the downregulation of MARCO expression, cytokine secretion decreased **(E-I)** and the inhibition of Nr-CWS on HeLa cells proliferation decreased **(J)**. n = 3, ^*^P < 0.05, ^**^P < 0.01, ^***^P < 0.001.

To investigate the role of MARCO in Nr-CWS-enhanced phagocytosis in TAMs, we silenced MARCO expression using small interfering RNA (siRNA) (**Supplementary Figure 13**). Neutral Red Uptake Assay showed that phagocytic activity was significantly reduced following MARCO knockdown (**Figure 8A**, P < 0.05). Additionally, fluorescence microscopy revealed a significant decrease of F-actin expression in pseudopodia after siRNA-mediated inhibition of MARCO, accompanied by a redistribution of the cytoskeleton from pseudopodia protrusions to the submembrane region (**Figure 8B**). Furthermore, phagosome acidification was notably diminished upon MARCO suppression (**Figures 8C, D**, P < 0.05). Together, these results strongly support the essential role of MARCO mediating the phagocytic activity of TAMs in response to Nr-CWS treatment.

Similar to its role in phagocytosis, we proposed that MARCO might also be a key mediator in Nr-CWS-induced cytokine secretion by TAMs. To test this, we assessed the secretion levels of IL-1α, IL-1β, IL-15, TNFα and IL-10 from TAMs following the inhibition of MARCO expression. ELISA results indicated that MARCO knockdown significantly reduced the secretion of pro-inflammatory cytokines (**Figures 8E–H**, all *P* < 0.05), while IL-10 levels remained unchanged (**Figure 8I**, P < 0.05). This suggests that MARCO may specifically mediate the Nr-CWS-induced release of pro-inflammatory cytokines from TAMs. Similarly, the anti-proliferative effect of Nr-CWS on HeLa cells was attenuated in TAMs with MARCO silencing (**Figure 8J**, P < 0.05). Together, these findings indicate that MARCO contributes to both the pro-inflammatory cytokines secretion by TAMs and their ability to inhibit HeLa cell proliferation.

### 2.10 A preliminary exploration of the potential role of toll-like receptor 4 in MARCO-mediated recognition of Nr-CWS

**Figure 9:**
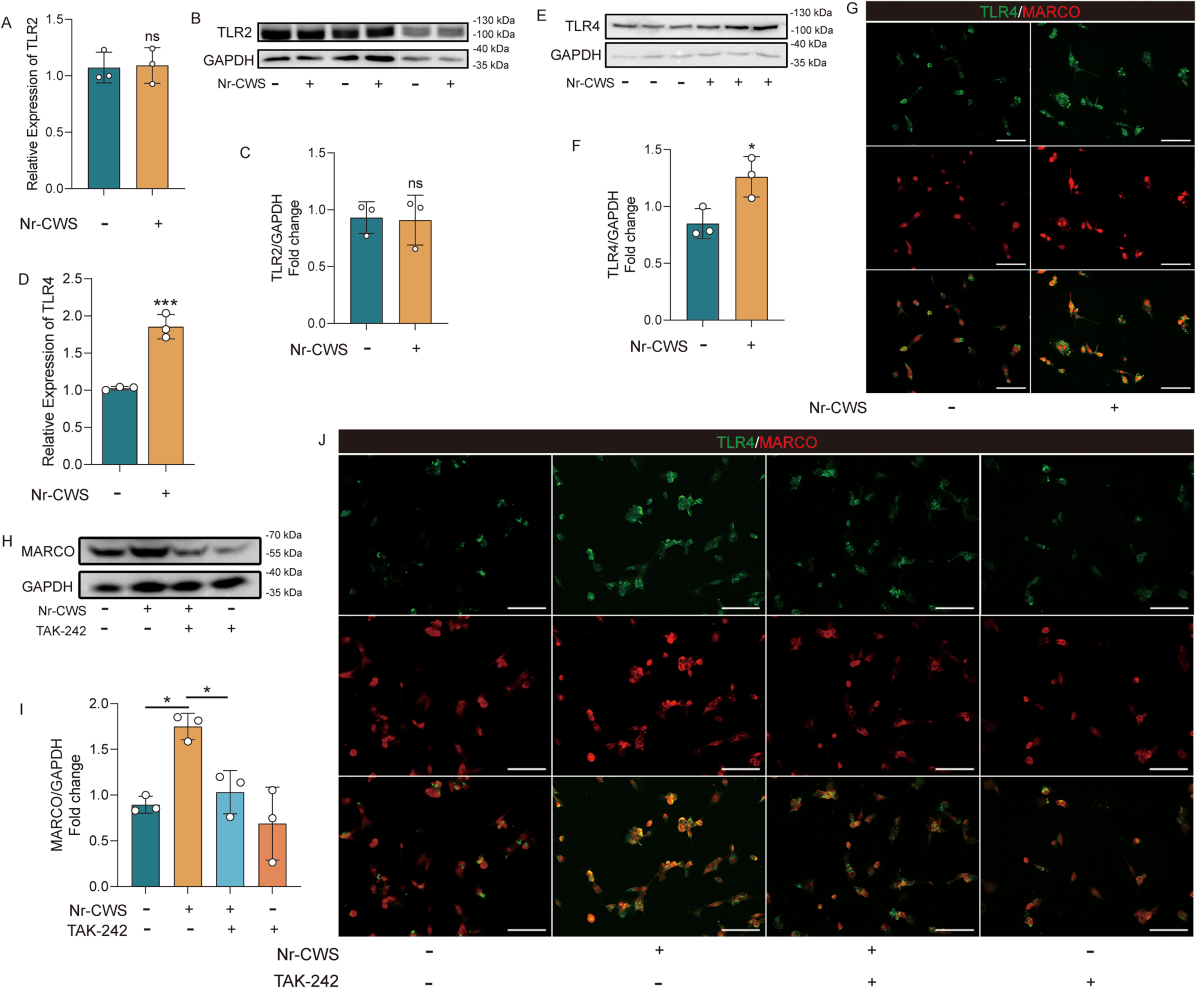
Nr-CWS affects MARCO expression through TLR4. The expression of TLR4 significantly increased after Nr-CWS treatment [**(D-G)**, scale bars = 100 μm], while the expression TLR2 was unchanged **(A-C)**. Subsequent inhibition of TLR4 expression resulted in decreased levels of MARCO [**(H-J)**, scale bars = 100 μm]. n = 3, ^*^P < 0.05, ^**^P < 0.01, ^***^P < 0.001.

Previous research has demonstrated that toll-like receptors (TLRs) can recognize specific molecular patterns and are closely associated with the regulation of phagocytic receptors. However, our RT-qPCR and WB analyses indicate a significant upregulation of toll-like receptor 4 (TLR4) -but not toll-like receptor 2 (TLR2)-following Nr-CWS treatment(**Figures 9A–F**, all *P* < 0.05). This suggests that TLR4 may be involved in mediating the immunomodulatory effects of Nr-CWS. We also performed double immunofluorescence labeling to assess TLR4 and MARCO expression. The findings indicated that after Nr-CWS treatment, both MARCO and TLR4 levels were elevated, and their co-localization increased significantly (**Figure 9G**). We therefore hypothesize that some form of connection between MARCO and TLR4 might exist in the context of Nr-CWS treatment. To test this hypothesis, we next used TLR4-specific inhibitor resatorvid (TAK-242) to suppress TLR4 expression in TAMs. Western blot analysis revealed a significant decrease in MARCO expression following TLR4 inhibition (**Figures 9H, I**, P < 0.05). Double Immunofluorescence labeling analysis further showed a reduced expression of both MARCO and TLR4, along with diminished co-localization of the two proteins after TLR4 suppression (**Figure 9J**). limited indications that TLR4 might be associated with Nr-CWS-induced upregulation of MARCO expression. However, any definitive conclusion regarding the relationship between TLR4 and MARCO would require additional experimental validation. The precise mechanistic role of TLR4 in this process remains largely unclear and warrants in-depth investigation.

## 3 Discussion

According to the 2022 global cancer statistics published by the International Agency for Research on Cancer (IARC) in 2024, cervical cancer ranks as the fourth most prevalent cancer in terms of both incidence and mortality among women. It is the second most frequent malignancy of the female reproductive system, following breast cancer (24). Notably, the average age of onset for cervical cancer is progressively declining, posing a significant threat to women’s health.

In recent years, the tumor microenvironment (TME) has gained increasing recognition for its critical role in tumor initiation, progression, and prognosis (25, 26), making it a key focus in anti-tumor therapeutic development (27). Furthermore, unlike conventional therapies that often encounter tumor cells resistance, immunomodulatory strategies targeting the TME tend to exhibit greater stability and specificity, which can enhance treatment efficacy while reducing adverse effects on patients.

Nr-CWS, as an immunomodulator, has been widely used in the clinical treatment of HR-HPV infections. Numerous studies have confirmed its significant therapeutic efficacy and its ability to activate the immune response within the cervical microenvironment. Since 2019, we have monitored the clearance of HPV infection in 84 subjects and investigated the potential restorative effects of Nr-CWS on the abnormal cervical tissue structure resulting from HPV infection using hematoxylin and eosin (H&E) staining. Our findings indicate that Nr-CWS contributes to the structural restoration of cervical tissue in HPV infected patients.

To further investigate the specific immunomodulatory mechanisms of Nr-CWS in clearing HPV infection, we selected three patients who tested negative after Nr-CWS treatment, and analyzed their cervical tissues before and after therapy using gene chip technology. Functional enrichment analysis of up-regulated genes revealed that MARCO was the enrichment gene of the term “endocytic vesicle membrane cell components” and the term “phagosome signaling pathway” that are first enriched in Gene Ontology (GO) and Kyoto Encyclopedia of Genes and Genomes (KEGG), respectively.

MARCO, one of the pattern recognition receptors on the surface of macrophages, is a member of the class A scavenger receptor family. In this study, analysis of TCGA data revealed differential MARCO expression in multiple tumor types compared to normal tissues. For example, MARCO expression was significantly down-regulated in tumor tissues such as CHOL, liver hepatocellular carcinoma (LIHC), lung adenocarcinoma (LUAD), lung squamous cell carcinoma (LUSC), and pheochromocytoma and paraganglioma (PCPG), whereas it was upregulated in tumor tissues such as KIRC, kidney renal papillary cell carcinoma (KIRP), and thyroid cancer (THCA). Besides, we explored the relationship between MARCO expression and patient prognosis, finding that its prognostic significance varies across different cancers. Moreover, the high expression of MARCO in Cervical Squamous Cell Carcinoma (CESC) is associated with poor prognosis, indicating its importance in cervical-related cancers. These elevated MARCO had independent prognostic value in clinical tumor treatment.

Tumor-infiltrating immune cells are closely related to tumor progression and patient prognosis (28). Understanding the relationship between MARCO and these immune cells could provide insight into its role in tumor development. Therefore, using the data from TIMER and TISIDB databases, we analyzed the correlation between MARCO expression and immune cells infiltration across various tumors. We found that MARCO is significantly associated with macrophage infiltration in most tumor types. Previous studies have shown that MARCO is expressed in specific macrophage subsets present in the spleen, lymph nodes, and lungs (17, 29). Macrophages play a critical role in clearing pathogens, and their functional plasticity allows them to adopt distinct phenotypes in response to signals from the surrounding immune microenvironment (26, 30–32). This functional heterogeneity may help explain why the association between MARCO expression and prognosis differs across tumor types.

Our findings demonstrate a significant upregulation of MARCO expression in cervical tissues from the same patient group following Nr-CWS treatment, compared to pre-treatment baseline levels. Unlike the constitutive overexpression or suppression of MARCO reported in established malignancies, our data reveal dynamic, time-dependent modulation of MARCO in response to Nr-CWS therapy in HPV-infected cervical lesions. We propose that this pharmacologically induced MARCO expression may represent a previously unrecognized immunomodulatory mechanism of Nr-CWS, highlighting its dual relevance in antiviral and anti-tumor immunity. Notably, this finding presents an intriguing contrast to recent therapeutic strategies in oncology that focus on blocking MARCO to suppress its pro-tumorigenic functions within the tumor microenvironment. We hypothesize that in the setting of HPV-associated precancerous lesions, Nr-CWS may engage MARCO as part of a broader pathogen recognition and macrophage activation program, steering TAMs toward an anti-tumor phenotype. This is mechanistically distinct from scenarios in established tumors where chronically engaged MARCO might contribute to an immunosuppressive niche. Prior studies have established that MARCO is predominantly expressed in specific macrophage subsets (33). This is consistent with the modern paradigm that TAMs represent a highly plastic and transcriptionally diverse population, a heterogeneity that is crucial for determining tumor outcomes (34). It has been observed that certain TAMs subpopulations adjacent to the tumor nest express MARCO. Our earlier work showed that Nr-CWS treatment reduces the proportion of M2-type macrophages while increasing that of M1-type macrophages in cervical tissues (35). Moreover, this finding of an M1/M2 phenotypic shift was further confirmed by our *in vitro* immunofluorescence double-staining assays. This finding suggests that Nr-CWS effectively activates TAMs within the tumor microenvironment, reprograms them towards an anti-tumor phenotype. This M1 polarization, coupled with the observed MARCO upregulation, aligns with the concept that the functional outcome of MARCO signaling is not intrinsic but is determined by the immunological context and the nature of its ligands. The components of Nr-CWS likely act as novel ligands or co-stimuli that convert MARCO into a node for immune activation rather than suppression. Integrating these findings with bioinformatics analyses, we hypothesize that the upregulation of MARCO in cervical tissues of patients following Nr-CWS treatment is mechanistically linked to the enhanced anti-tumor function of TAMs. To test this, we initially examined the co-localization of MARCO and the macrophage marker CD68 in cervical tissues post Nr-CWS treatment using double immunofluorescence labeling staining. The findings indicated that Nr-CWS predominantly stimulated macrophages within the tumor microenvironment to express MARCO. Subsequent *in vitro* experiments confirmed that the induced MARCO expression strongly colocalized with the M1 marker CD86, indicating its specific association with M1-polarized TAMs. Additionally, siRNA-mediated knockdown of MARCO in TAMs allowed us to assess its functional necessity, notably in phagocytic activity and proinflammatory cytokines secretion.

Macrophages, as primary phagocytes, are critical for pathogen recognition and clearance (36). Previous studies indicates that Nr-CWS enhances macrophage phagocytosis of fluorescent microspheres and dextran particles (37, 38). Phagocytosis involves cytoskeletal reorganization, which elongates macrophage protrusions to engulf pathogens via endocytosis, forming phagosomes. These phagosomes subsequently fuse with lysosomes, leading to pathogen degradation and elimination. Initially, we examined the morphological alterations in macrophages after Nr-CWS treatment using electron microscopy, and the observations suggested a potential effect on macrophage phagocytosis. Furthermore, we newly found that Nr-CWS enhances the production and reorganization of the macrophage cytoskeletal protein F-actin, promotes lysosome production and augments the macrophage-mediated phagocytosis and killing of HeLa cells. These results further confirm the conclusion that Nr-CWS enhances the macrophage phagocytosis function. To directly assess the role of MARCO in this process, we evaluated the impact of MARCO deletion on macrophage phagocytosis. MARCO, a phagocytic receptor expressed on the macrophage surface, is closely associated with phagocytic capacity (39–43). Inhibition of MARCO expression led to a reduction in Nr-CWS-enhanced phagocytosis in TAMs, accompanied by decreased F-actin expression in TAMs pseudopods, cytoskeletal remodeling, inhibited stress fiber formation, and significantly reduced phagosome acidification. Our findings demonstrate that MARCO inhibition impairs the phagocytic function of TAMs. Collectively, these findings show that Nr-CWS can enhance the phagocytic function of TAMs, and that MARCO on macrophages represents a promising therapeutic target for t HPV infection. This “activation” strategy, centered on inducing a specific phagocytic receptor, offers a distinct yet complementary approach to current therapeutic paradigms that aim to deplete or inhibit immunosuppressive TAM subpopulations.

Additionally, macrophages modulate the tumor immune microenvironment through cytokine secretion, influencing adaptive immune response and directly affecting tumor cell activity (44–46). To investigate whether Nr-CWS affects this secretory function, we measured the levels of four pro-inflammatory cytokines-IL-1α, IL-1β, TNFα, and IL-15- in culture supernatants. These cytokines are well-established markers commonly used to assess the pro-inflammatory function of M1-polarized macrophages and are potentially linked to both Nr-CWS and MARCO. IL-1β and TNFα are extensively studied pro-inflammatory cytokines known for their roles in sustaining and amplifying inflammatory responses, modulating the function of various immune cells, participating in antiviral immune responses, and exerting cytotoxic effects on tumor cells. Our study showed that Nr-CWS treatment significantly enhance the secretion of well-characterized pro-inflammatory cytokines IL-1β and TNFα. Furthermore, we made a novel observation that Nr-CWS also increased the secretion of IL-1α and IL-15. The induction of IL-15 is of particular mechanistic and translational significance. As a pivotal γc-chain cytokine, IL-15 is of particular interest as a significant target in tumor immunotherapy due to its ability to bridge innate and adaptive immunity by stimulating T-cell and natural killer (NK) cell proliferation, differentiation and activation. Our finding that Nr-CWS upregulates macrophage-derived IL-15 provides a plausible mechanistic link to prior reports that Nr-CWS facilitates NK cell differentiation and augments their cytotoxic activity Given previous evidence that Nr-CWS promotes NK cell differentiation and cytotoxicity (47), we hypothesize that the upregulation of IL-15 secretion may be a key mechanism underlying this effect, a conjecture warranting future investigation. We hypothesize that Nr-CWS, through activating macrophages and inducing MARCO, establishes a pro-inflammatory niche characterized by elevated IL-15. This cytokine milieu could act in a paracrine manner to sustain and activate neighboring NK cells and T cells within the tumor microenvironment, thereby orchestrating a more potent and coordinated anti-tumor response. This potential axis-from Nr-CWS to MARCO^+^ macrophage activation, to IL-15 secretion, and finally to lymphocyte priming-represents a compelling multi-step immunomodulatory circuit that warrants rigorous future validation. We next investigated the role of MARCO in this cytokine response. Our studies further demonstrate that the enhanced secretion of IL-1α, IL-1β, TNFɑ and IL-15 induced by Nr-CWS is dependent on MARCO activation. This aligns with previous studies indicating that MARCO is closely associated with the secretion of cytokines by TAMs. And the interaction between ligands and MARCO can trigger intracellular signaling cascades, lead to the activation of transcriptional programs for genes involved in inflammation, and stimulate macrophages to secrete cytokines pertinent to inflammatory responses. In addition to pro-inflammatory cytokines, our preliminary investigation also examined the expression of anti-inflammatory cytokine IL-10. IL-10 acts as a key immunosuppressive cytokine that dampens inflammation and fosters an immunosuppressive tumor microenvironment, which ultimately promotes immune evasion and confers resistance to therapy. However, our data indicated that MARCO suppression did not significantly alter its level in macrophages, suggesting that Nr-CWS may enhance the secretion of pro-inflammatory cytokines, as opposed to anti-inflammatory ones, in macrophages. Collectively, the Nr-CWS-induced MARCO expression might be part of a coordinated cellular program that includes cytoskeletal remodeling and metabolic reprogramming, collectively enhancing the anti-pathogen and anti-tumor capacity of TAMs in the cervical microenvironment.

Furthermore, research has indicated that key components of Nr-CWS, such as peptidoglycan and arabinogalactan, can activate the Toll-like receptor (TLR)/Myeloid differentiation primary response gene 88 (MyD88) signaling pathway (48, 49). Previous research has demonstrated a significant association between TLRs and HPV infection, as well as the onset and progression of cervical lesions (50). Additionally, studies have indicated that TLR ligands can induce the expression of phagocytic proteins, including MARCO, thereby specifically enhancing bacterial phagocytosis in both murine and human cells (51, 52). Consequently, TLR signaling may constitute a critical upstream mechanism through which Nr-CWS upregulates MARCO expression and enhances macrophage function.

TLR family plays a pivotal role in innate immunity and inflammatory responses against pathogens, with TLR2 and TLR4 being among the most prominent members. Recent research has revealed a functional synergy between TLR2 and MARCO, demonstrating that their co-activation can augment MARCO-mediated inflammatory response (53–55). However, our study demonstrated that TLR2 expression remained unchanged after Nr-CWS treatment, whereas TLR4 expression exhibited a significant upregulation. This observation led us to hypothesize a potential role for TLR4, as opposed to TLR2, in the therapeutic process of Nr-CWS. To preliminarily assess this possibility, we employed the TLR4-specific inhibitor TAK-242 to suppress TLR4 expression. Following the inhibition of TLR4 expression, a concomitant decrease in both MARCO expression and co-localization of TLR4 and MARCO was observed, which appears to support the possibility of a functional link between TLR4 and MARCO during Nr-CWS treatment. It should be noted, however, that the present experimental strategy provides only initial insights. We will conduct further investigations into the relationship between TLR4 and MARCO in the context of Nr-CWS action, and do more rigorous, comprehensive validation such as genetic knockdown or knockout of TLR4, detailed analysis of downstream signaling pathways, and expanded sample sizes in future studies.

Overall, our findings demonstrate that MARCO is pivotal in augmenting the anti-tumor function of TAMs. This study specifically demonstrates that Nr-CWS modulates TAMs through the involvement of the scavenger receptor MARCO. We observed that MARCO activation is associated with key functional alterations in macrophages—namely, enhanced phagocytic capacity and a shift in cytokine secretion toward an immunostimulatory profile. These changes represent central downstream effects of MARCO engagement, directly linking Nr-CWS-induced MARCO modulation to tangible functional outcomes in TAMs. Mechanistically, we propose that TLR4 acts as an integral component in this process, potentially serving as a recognition receptor for Nr-CWS and subsequently inducing MARCO upregulation, which may in turn initiate a cascade of downstream molecular alterations. Although the complete upstream regulatory mechanism of MARCO expression by Nr-CWS remains to be fully elucidated and warrants further investigation, our data provide important insights into its functional consequences. Collectively, this study offers a clearer mechanistic narrative for how Nr-CWS may reshape the tumor immune microenvironment in cervical cancer, highlighting the potential of pharmacologically targeting MARCO for the treatment of HPV infection and cervical precancerous lesions. Future studies delineating the precise upstream sensors of Nr-CWS and its impact on the metabolic landscape and transcriptional profile of TAMs will further elucidate this promising immunomodulatory mechanism.

## 4 Materials and methods

### 4.1 Chemicals and antibodies

Nr-CWS (S20030009) was obtained from Weihai Greatest Pharmaceutical Research Institute Co., Ltd. (China). Phorbol 12-myristate 13-acetate (PAM, Cayman Chemical, 10008014, USA) was dissolved in sterile dimethyl sulfoxide (DMSO, Solarbio Life Sciences, D8671, China) as a 1 mg/mL stock solution. Neutral red (Guangfu Fine Chemical, China) and trypan blue (Solarbio Life Science, C0040, China) were dissolved in normal saline solution to create stock solutions of 4 mg/mL, respectively, and were subsequently filtered through a 0.22-μm filter. TAK-242 (Cayman Chemical, 13871, USA), a toll-like receptor 4 (TLR4)-specific inhibitor, was dissolved in sterile DMSO as a 5 mM stock solution. Carboxyfluorescein diacetate, succinimidyl ester (CFDA-SE, Cayman Chemical, 14456, USA), a stable cell-permeable dye, was dissolved in sterile DMSO as a 10 mM stock solution. Phalloidin-Fluorescein Conjugate (Cayman Chemical, 20478, USA), which selectively binds to filamentous actin (F-actin), was solubilized in DMSO. LysoTracker^®^ Red DND-99 (Invitrogen, L7528, USA), a fluorescent probe used for labeling and tracking acidic organelles in live cells, was dissolved in anhydrous DMSO as a 1 mM stock solution. Neutral red and trypan blue solutions were stored at 4 °C, while others were stored at -80 °C from light by foil.

The following commercial antibodies (vendor, catalog number and dilution) utilized for western blot and immunohistochemical staining were employed in accordance with the manufacturers’ guidelines: mouse anti-CD68 antibody (Abcam, ab201340, 1:200), rabbit anti-MARCO antibody (Abcam, ab231046, 1:1000 for western blot and 1:100 for immunohistochemistry), rabbit anti-CD163 antibody (Abways Technology, CY6845, 1:500), mouse anti-CD86 antibody (Proteintech, 68674-2-Ig, 1:5000), mouse anti-TLR4 antibody (Santa Cruz Biotechnology, sc-293072, 1:1000 for western blot and 1:200 for immunohistochemistry), rabbit anti-TLR2 antibody(Abways Technology, CY5102, 1:2000), mouse anti-GAPDH antibody (Proteintech, 6004-1-Ig, 1:50000), HRP-goat anti-mouse recombinant secondary antibody (H+L)(Proteintech, RGAM001, 1:5000), goat anti-mouse IgG (H+L) Alexa Fluor 594 (Abways Technology, AB0152, 1:300), HRP-goat anti-rabbit recombinant secondary antibody (H+L) (Proteintech, RGAR001, 1:5000), goat anti-rabbit IgG (H+L) secondary antibody DyLight™ 594 (Report Biotech, S7002, 1:500), goat anti-mouse IgG (H+L) secondary antibody DyLight™ 488 (Report Biotech, S6001, 1:500), goat anti-mouse IgG (H+L) secondary antibody DyLight™ 594 (Abways Technology, AB0152, 1:500).

### 4.2 Patients

A cohort of 84 patients, aged 21 to 65 years, were selected from the Fourth Hospital of Hebei Medical University, China. The selection criteria were predetermined, and all patients underwent treatment with Nr-CWS (13). Cervical tissue samples were obtained from participants in the CIN group prior to topical administration of Nr-CWS, as well as 30 or 90 days post-administration, respectively.

Informed consent of this study was obtained from all participants and the ethical approval was granted by the Ethics Committees of The Fourth Hospital of Hebei Medical University (2019MEC097).

### 4.3 Paraffin section and H&E staining

Tissue samples were initially fixed in 4%(w/w) paraformaldehyde for a period of 24 hours, followed by dehydration and embedding in paraffin. Subsequently, tissues were sectioned into 3.5 μm-thick slices using a slicer, and stained with hematoxylin and eosin (H&E) and observed using a microscope camera (Olympus, Japan).

### 4.4 Gene expression microarray analysis

#### 4.4.1 Differential expression analysis in cervical tissues post Nr-CWS treatment

The gene chip technology was performed on cervical tissues pre- and post-Nr-CWS treatment of three patients with CIN. Total RNA extracted from cervical tissues using the miRNeasy Mini Kit was subjected to gene expression profiling with Affymetrix Clarion S Human arrays. Following the recommended protocol, amplified and biotin-labeled cDNA was prepared, hybridized to the arrays, and scanned. The resulting CEL files were processed with the RMA algorithm for background correction and normalization in the R environment. The R package “limma” was used for differential expression analysis. Differentially expressed genes between pre- and post-Nr-CWS treatment groups were defined as the upregulated or down-regulated genes with a fold change ≥ |2| and *P* < 0.05. In addition, expression levels were visualized as heatmaps using the pheatmap package and we mapped the volcano plot using ggplot2 package in R software.

#### 4.4.2 GO and KEGG enrichment analysis

Functional enrichment analysis includes Gene Ontology (GO) and Kyoto Encyclopedia of Genes and Genomes (KEGG) in this study. In brief, GO enrichment analyses predict the function of the target genes and KEGG is a widely used database for systematic signaling pathway analysis according to gene functions. In this study, the clusterProfiler package in R software was used to perform the functional enrichment analysis and ggplot2 package in R software was employed to visualize all of the enrichment analysis results.

### 4.5 MARCO expression, survival and immune cell infiltration analysis

We investigated the differential expression of MARCO between tumor and adjacent normal tissues across all TCGA tumors in TIMER2.0 (56–58). And we used the GSCA database (59, 60) to search for a link between the expression level of MARCO and survival significance, such as OS and DSS. The relative abundance of the tumor infiltrating lymphocytes (TILs) in cancers with different MARCO mRNA expression statuses was calculated by TIMER2.0 and TISIDB (61).

### 4.6 Cell preparations

#### 4.6.1 Cell culture and supernatant collection

HeLa and THP-1 cells were cultured in RPMI 1640 medium (Gibco, C11875500BT, USA) supplemented with 1% penicillin-streptomycin liquid (Solarbio Life Sciences, P1400, China) and 10% fetal bovine serum (FBS, Corning, 35-081-CV, USA). The cells were maintained in a controlled environment at 37 °C with 5% CO_2_.

HeLa cells underwent three passages before seeding. Cells were seeded at a density of approximately 10^4^ cells/cm^2^. After 48 h of culture, cells were with fresh RPMI 1640 media for a subsequent 48 h. Cell culture supernatant was collected by centrifuge and stored at -20°C before use.

#### 4.6.2 Cell induction and cultivation

THP-1 cells were treated with 20 ng/mL PMA at a concentration of 10^6^ cells/mL for a duration of 48 hours to promote the differentiation into macrophages.

*In vitro* model of TAMs was established by polarizing PMA-induced M0 macrophages with HeLa-conditioned medium for a period of 2 days, confirming a CD68+CD163+ M2-like phenotype as a baseline for subsequent Nr-CWS treatment experiments (62–65).

#### 4.6.3 Co‐culture experiments

HeLa cells were labeled with CFDA-SE living cell dye according to the manufacturer ‘s instructions. HeLa cells were resuspended in RPMI 1640 media at a concentration of 10^7^ cells/mL. A volume of 1 μL of 10 mM CFDA-SE was added per 1 mL of cell suspension. After a 10-minute incubation at 37°C, cells were washed thrice and resuspended.

The co-culture of TAMs and HeLa cells was conducted to investigate the possible response between the two cells. Specifically, for 10^5^ per well TAMs, 5×10^4^ HeLa cells were added and incubated in medium for 48 hours. Subsequently, cell morphology was examined using scanning electron microscopy. Similarly, added CFDA-SE-labeled HeLa cells and confirmed through fluorescence microscopy.

### 4.7 Immunohistochemistry and immunocytochemistry

Immunocytochemistry was performed using mature inactive macrophages and immunohistochemistry was performed in tissues samples. Tissue sections subjected to overnight incubation with anti-MARCO primary antibodies while cells with anti-CD68. Immunohistochemical and immunocytochemical analysis was carried out using a suitable goat anti-rabbit or anti-mouse Immunohistochemical detection reagent kit (ZsBio, Beijing, China) as per the manufacturer’s instructions.

### 4.8 Immunofluorescence staining

Immunofluorescence was employed for the analysis of patient specimens and cells. Specifically, for double immunofluorescence (DIF), tissue slices were incubated with two first antibodies at the same time, rabbit anti-MARCO and mouse anti-CD68, while cells were exposed to rabbit anti-CD163 and mouse anti-CD68, rabbit anti-CD163 and mouse anti-CD86, rabbit anti-MARCO and mouse anti-CD86 or rabbit anti-MARCO and mouse anti-TLR4. Subsequently, goat anti-mouse IgG (H+L) DyLight™ 488, goat anti-mouse IgG (H+L) DyLight™ 594, goat anti- rabbit IgG (H+L) DyLight™ 488 and goat anti-rabbit IgG (H+L) DyLight™ 594 secondary antibody were utilized. For TAMs-HeLa co-culture model, cells were incubated with mouse anti-CD68 primary antibody and goat anti-mouse IgG (H+L) Alexa Fluor 594 secondary antibodies. Tissue sections and cells were then fixed with Prolong Gold Antifade Reagent with 4’,6-Diamidino-2’-phenylindole (DAPI), and observed using laser confocal microscopy (Olympus, Japan) after mounting.

### 4.9 SEM

The samples were fixed in a 2.5% glutaraldehyde buffer for 30 minutes followed by dehydration through an alcohol gradient. The samples were dried with tert-butanol, coated with gold particles, and examined under a scanning electron microscope (Regulus8100, Japan).

### 4.10 Reverse transcription quantitative polymerase chain reaction

Total RNA was extracted utilizing the TRIpure Reagent (Aidlab Biotechnologies, RN01, China), followed by complementary DNA (cDNA) synthesis using the PrimeScript™ RT reagent Kit (TaKaRa, RR047A, Japan). Subsequently, quantitative polymerase chain reaction (qPCR) was conducted on the QuantStudio3 (Applied Biosystems, USA) platform, with amplification carried out using the TB Green^®^ Premix Ex Taq™ II (TaKaRa, RR820A, Japan). The reactions were normalized to glyceraldehyde-3-phosphate dehydrogenase (GAPDH) and the primers used in this study are listed in **Supplementary Table 1**.

### 4.11 Western blot analysis

Cells were harvested and lysed in radio immunoprecipitation assay (RIPA) buffer (Seven Biotech, SW104, China) supplemented with a protease inhibitor cocktail (MCE, HY-K0010, USA). The lysates were then mixed with sodium dodecyl sulfate (SDS) loading buffer (Solarbio Life Sciences, P1040, China) and heated at 100 °C for 5 minutes. After measuring the protein concentration, equal amounts of protein were subjected to sodium dodecyl sulphate-polyacrylamide gel electrophoresis (Seven Biotech, SW109-01, China). Transferring the protein onto polyvinyl difluoride membranes (Millipore, USA), blocking with 5% dry skim milk, and subsequently incubating at 4 °C overnight with first antibodies targeting MARCO, TLR4, and GAPDH. Following this, the membranes were exposed to recombinant secondary antibodies for 2 hours. Detecting protein bands with an enhanced chemiluminescence (ECL) kit (Biosharp Life Sciences, BL523B, China) and capturing with an Amersham Imager 600 (GE Healthcare, Pittsburgh, PA, USA). The relative protein expression levels were determined by normalizing to GAPDH.

### 4.12 Phagocytosis assay

#### 4.12.1 Neutral red uptake assay

Macrophages at a concentration of 10^6^ cells/mL were treated with 7.5 μg/ml of Nr-CWS for 48 hours. Subsequently, cells were exposed to 100 μL of a 0.1% (w/w) neutral red solution for 2 hours. Following the incubation period, cells were then lysed on the shaker for 20 minutes using 150 μL of a cell lysis buffer (Vethanol: Vacetic acid = 1:1). The absorbance of neutral red was quantified using a microplate reader (BioTek, EPOCH, USA).


$$Neutral\;red\;uptake\;rate\;(\%)=\frac{A_{s}}{A_{c}}\times 100$$

where

$A_{s}$ is the absorbance of treatment group.

$A_{c}$ is the absorbance of control group.

#### 4.12.2 Trypan blue uptake assay

One method employed to assess macrophage phagocytosis involved observing the uptake of trypan blue particles by cells (66–68). Trypan blue particles were added to the macrophage monolayers, which were then incubated for 48 hours to allow for internalization. Following incubation, cells were washed and subsequently fixed in 4% formaldehyde and examined under a microscope.

#### 4.12.3 Cytoskeleton staining

Cells were fixed with 4% paraformaldehyde, permeabilized with 0.5% Triton X-100 and then stained with phalloidin-fluorescein conjugate at room temperature in the dark for 30 min. Subsequently, cells were mounted on Prolong Gold Antifade Reagent with DAPI.

#### 4.12.4 Lysosome tracker assay

Phagosome acidification was evaluated by staining cells with 50 nM LysoTracker Red prior to fixation. Subsequently, the cells were permeabilized with 0.5% Triton X-100, followed by mounting on Prolong Gold Antifade Reagent with DAPI.

### 4.13 ELISA

According to the manufacturer’s instructions, IL-1α, IL-1β, IL-15, and TNFα expression levels in cell culture supernatant of TAMs were determined using IL-1α Human ELISA Kit (Abclonal, RK00031, China), IL-1β Human ELISA Kit (Abclonal, RK00001, China), IL-15 Human ELISA Kit (RK04454), and TNFα Human ELISA Kit (Abclonal, RK00030, China).

### 4.14 Small interfering RNAs

All siRNA oligonucleotides were synthesized by GenePharma (China). According to the manufacturer’s instructions, siRNAs were transfected using HighGene plus Transfection reagent (ABclonal Technology, RM09014P, China). Following transfection, the knock-down efficiency was assessed using RT-qPCR and Western blot analysis. The knock-down efficacy was quantified as the ratio of the target gene expression level normalized to GAPDH, following treatment with specific siRNA, divided by the corresponding value obtained with non-targeting siRNA treatment. The sequences are listed in **Supplementary Table 2**.

### 4.15 Cell counting kit-8 assay

HeLa cells, inoculated onto 96-well plates at a density of 5000 cells per well, were incubated for 48 hours with the cell culture supernatant of TAMs subjected to various treatments. Subsequently, the proliferation of HeLa cells was assessed using the Cell Counting Kit-8 (CCK-8) assay (Report Biotech, RK3028, China), with absorbance at 450 nm.


$$Cell\;viability\;(\%)=\frac{A_{s}}{A_{c}}\times 100$$

where

$A_{c}$ is the absorbance of untreated cells.

$A_{s}$ is the absorbance of Nr-CWS or MARCO siRNA treated cells.

### 4.16 Statistical analysis

Under the conventional sample size guidelines for similar studies, data from three randomly selected replicates were subjected to statistical analysis using SPSS 25.0 (SPSS Inc., USA), with significance determined through independent samples T-test or one-way analysis of variance. In bioinformatics analysis, R and corresponding R packages were utilized for statistical analysis. Student’s t-test was adopted for the analysis of continuous variables. Spearman’s correlation was performed to analyze correlations. At the same time, we performed survival analysis with Kaplan-Meier method, and survival was compared among multiple groups with the log-rank test. Significance was established at a P-value of less than 0.05, and data are expressed as mean ± standard deviation (SD).

## Data Availability

The datasets presented in this study can be found in online repositories. The names of the repository/repositories and accession number(s) can be found below: https://ngdc.cncb.ac.cn/omix, OMIX014924.
