# Supplementary material for: Nocardia rubra cell wall skeleton-induced MARCO expression: implications for improved phagocytosis and cytokine secretion in tumor-associated macrophages
Source: Front Immunol. 2026 Feb 12;17:1611476. doi: 10.3389/fimmu.2026.1611476 (PMC12936005; doi:10.3389/fimmu.2026.1611476)
Supplement: Supplementary file 1 [file Table1.docx]

Supplementary Material

# Supplementary Tables

**Supplementary Table 1.** Forward and reverse primer sequences.

| Primer name | Forward | Reverse |
| --- | --- | --- |
| MARCO | AGCACACCCTGGAGAACACC | CGCCTTGTTCACCTTTGATTCTGA |
| IL-1α | ACTCAGAGGAAGAAATCATCAAGC | CTCAGGCATCTCCTTCAGCAGCAC |
| IL-1β | GGATATGGAGCAACAAGTGG | ATGTACCAGTTGGGGAACTG |
| IL-15 | TTGGGAACCATAGATTTGTGCAG | GGGTGAACATCACTTTCCGTAT |
| TNFα | ATGAGCACTGAAAGCATGATCCGG | CTACAACATGGGCTACAGGCTTGT |
| TLR2 | CTCTCGGTGTCGGAATGTC | AGGATCAGCAGGAACAGAGC |
| TLR4 | ACCTGTCCCTGAACCCTAT | CTAAACCAGCCAGACCTTG |
| GAPDH | GTCTCCTCTGACTTCAACAGCG | ACCACCCTGTTGCTGTAGCCAA |

**Supplementary Table 2.** Small interfering RNA sequence.

| Name | Sense (5’- 3’) | Antisense (5’- 3’) |
| --- | --- | --- |
| MARCO siRNA 1 | AGCACACCCTGGAGAACACC | CGCCTTGTTCACCTTTGATTCTGA |
| MARCO siRNA 2 | ACTCAGAGGAAGAAATCATCAAGC | CTCAGGCATCTCCTTCAGCAGCAC |
| MARCO siRNA 3 | GGATATGGAGCAACAAGTGG | ATGTACCAGTTGGGGAACTG |
| MARCO siRNA 4 | TTGGGAACCATAGATTTGTGCAG | GGGTGAACATCACTTTCCGTAT |
| siNC | GTCTCCTCTGACTTCAACAGCG | ACCACCCTGTTGCTGTAGCCAA |

# Supplementary Figures


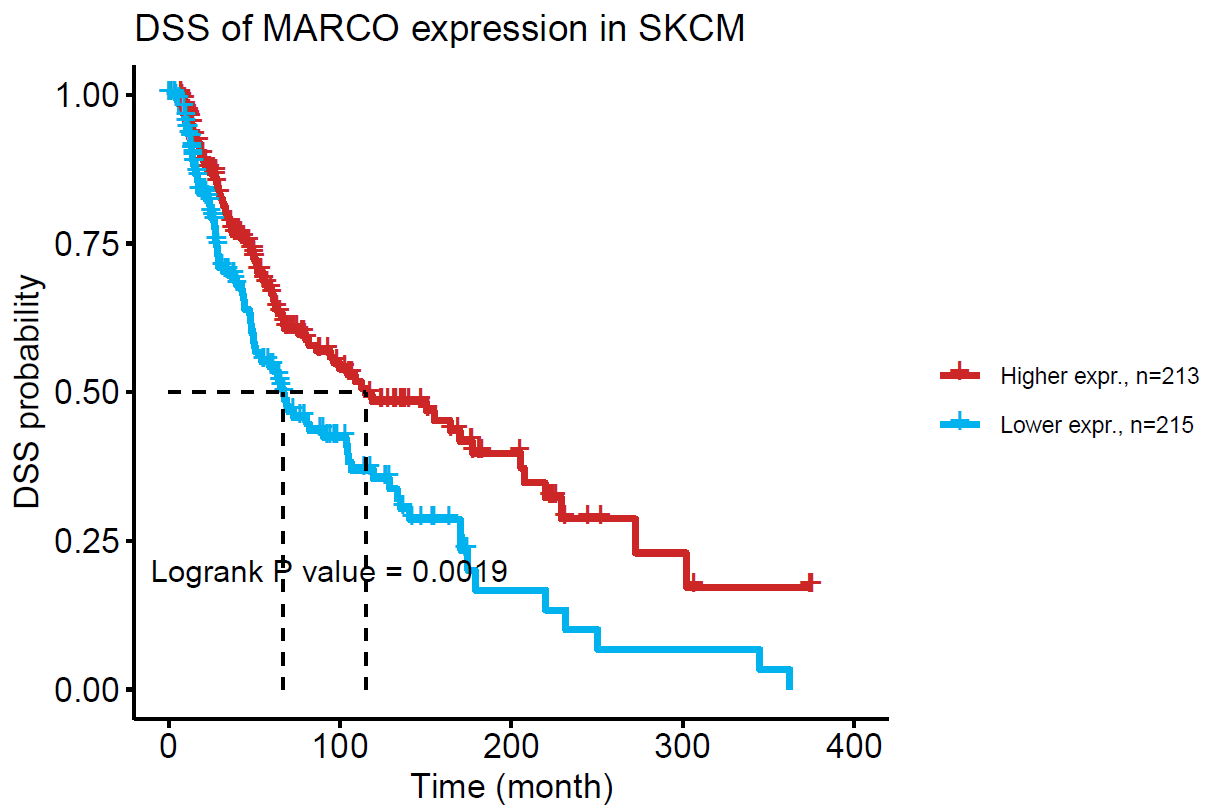


**Supplementary Figure 1.** DSS of MARCO expression in SKCM.


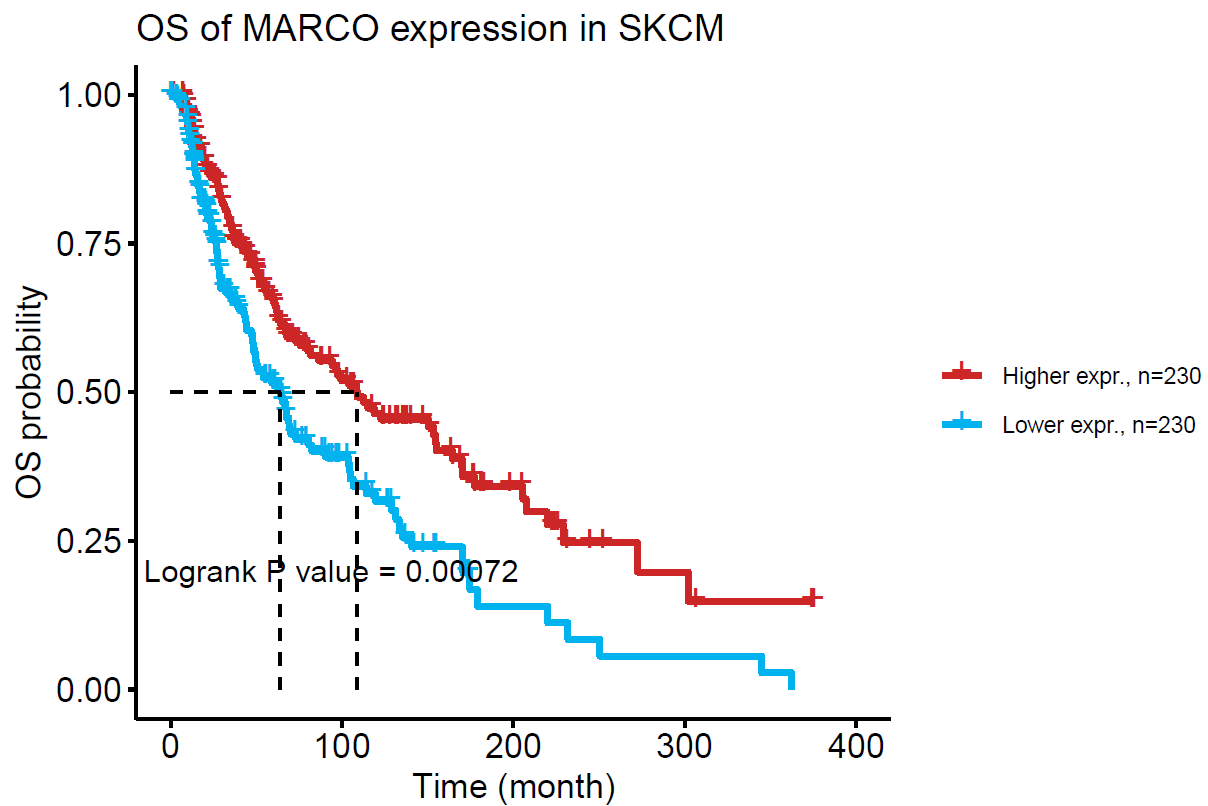


**Supplementary Figure 2.** OS of MARCO expression in SKCM.


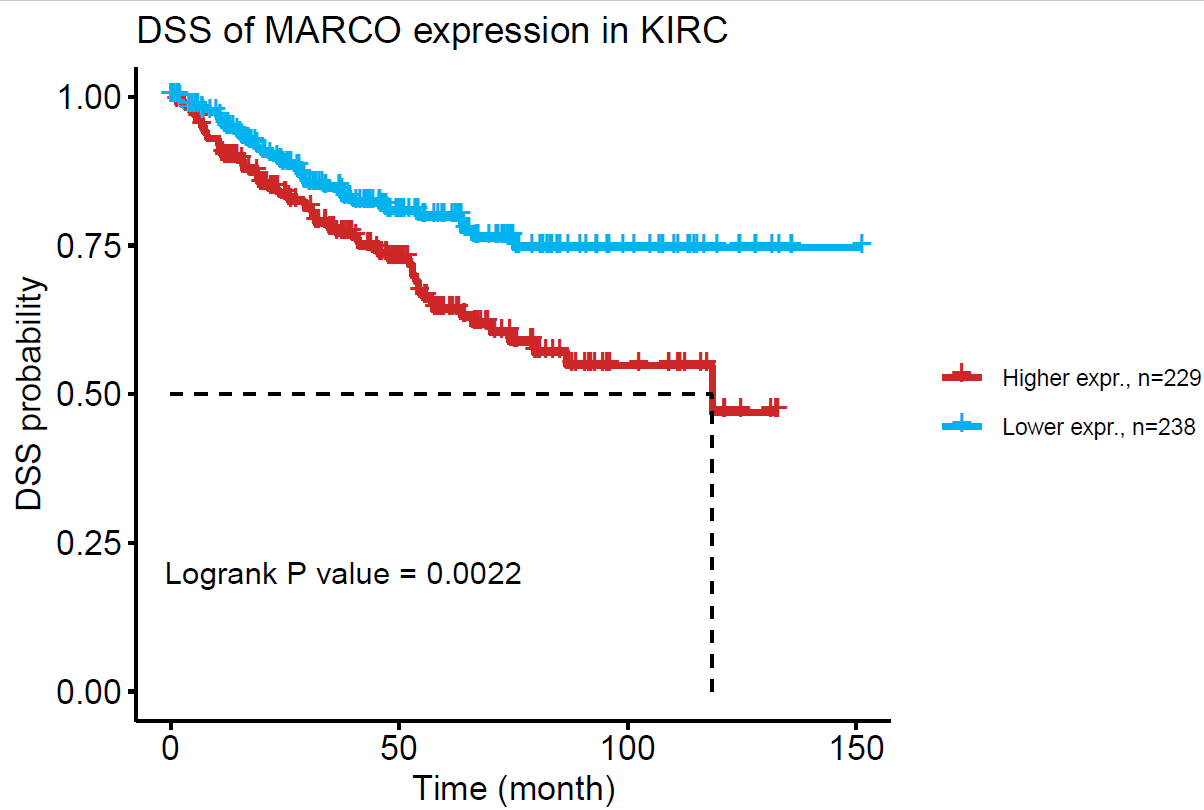


**Supplementary Figure 3.** DSS of MARCO expression in KIRC.


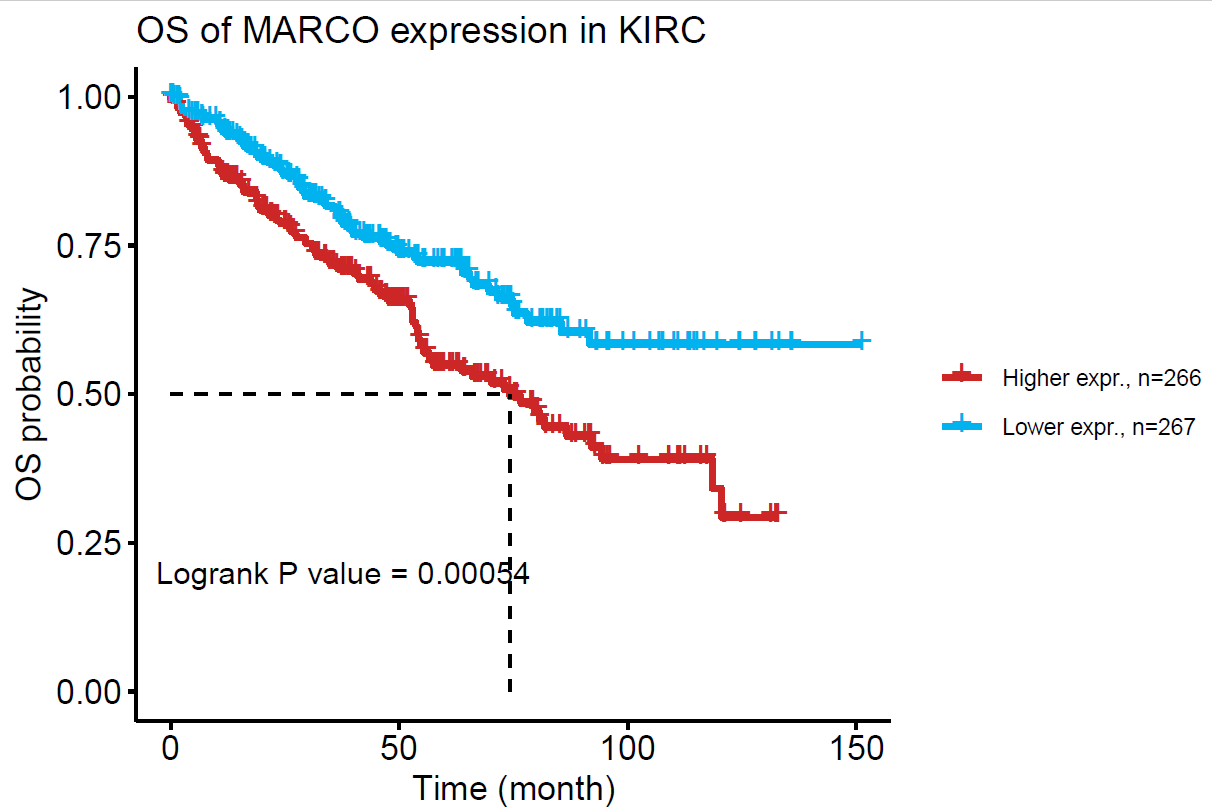


**Supplementary Figure 4.** OS of MARCO expression in KIRC.


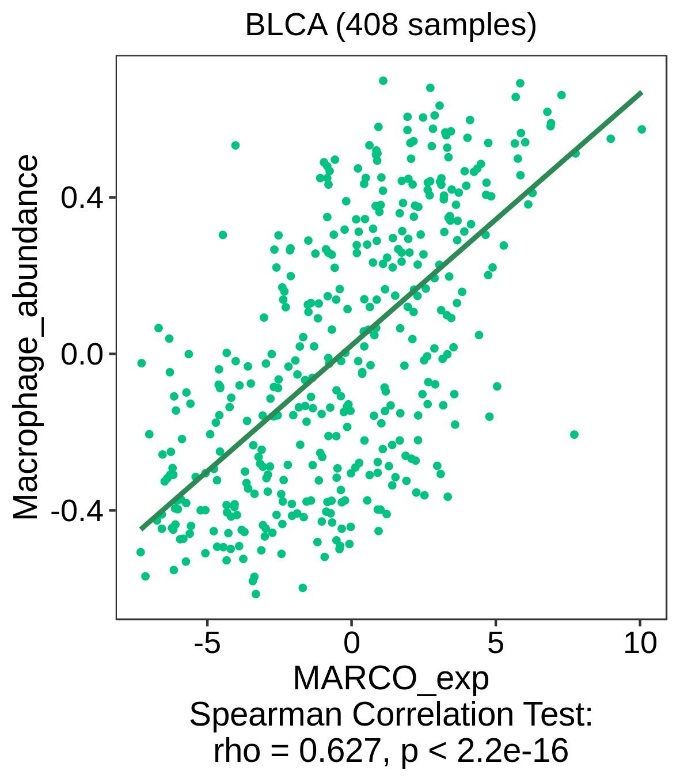


**Supplementary Figure 5.** The association between MARCO expression and macrophage infiltration in BLCA.


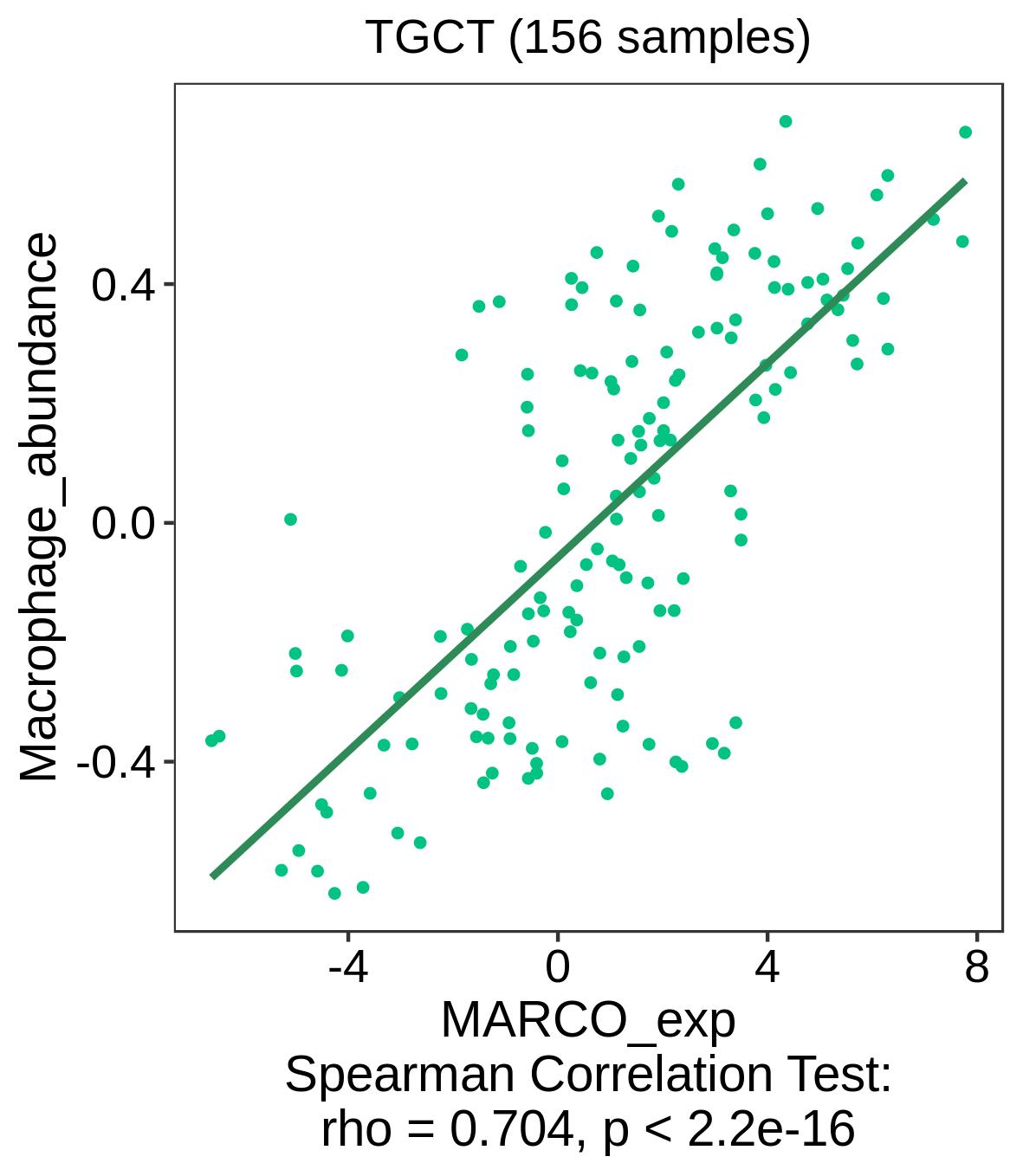


**Supplementary Figure 6.** The association between MARCO expression and macrophage infiltration in TGCT.


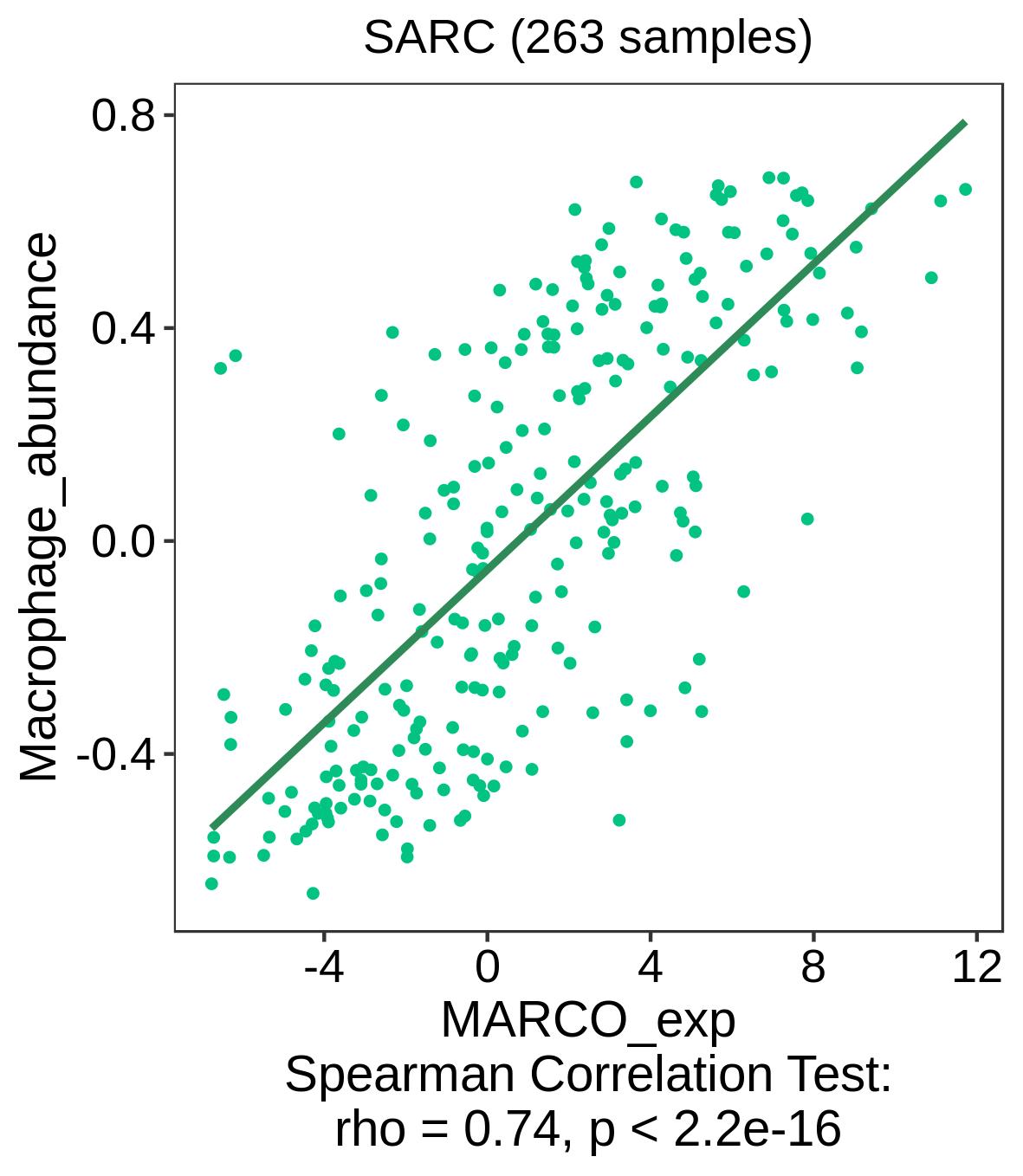


**Supplementary Figure 7.** The association between MARCO expression and macrophage infiltration in SARC.


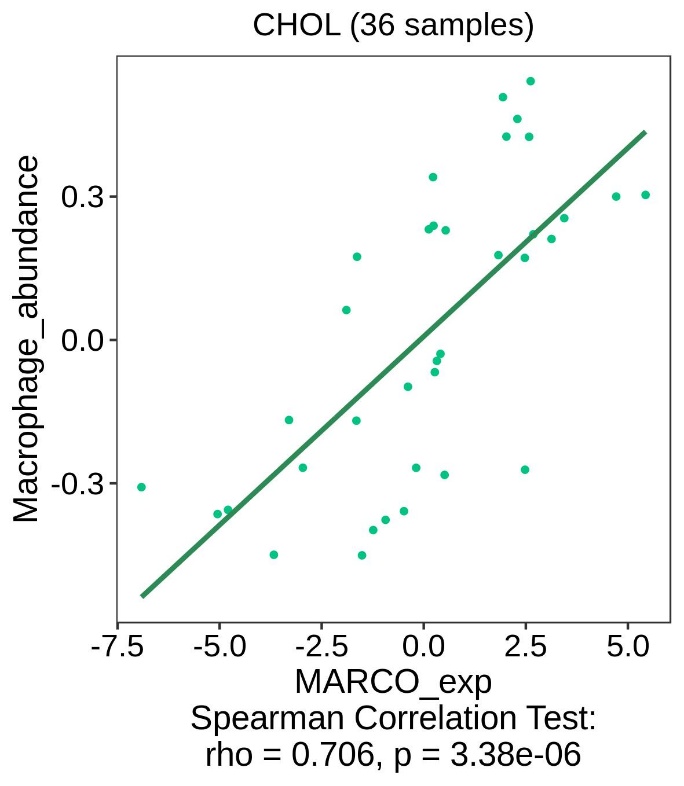


**Supplementary Figure 8.** The association between MARCO expression and macrophage infiltration in CHOL.


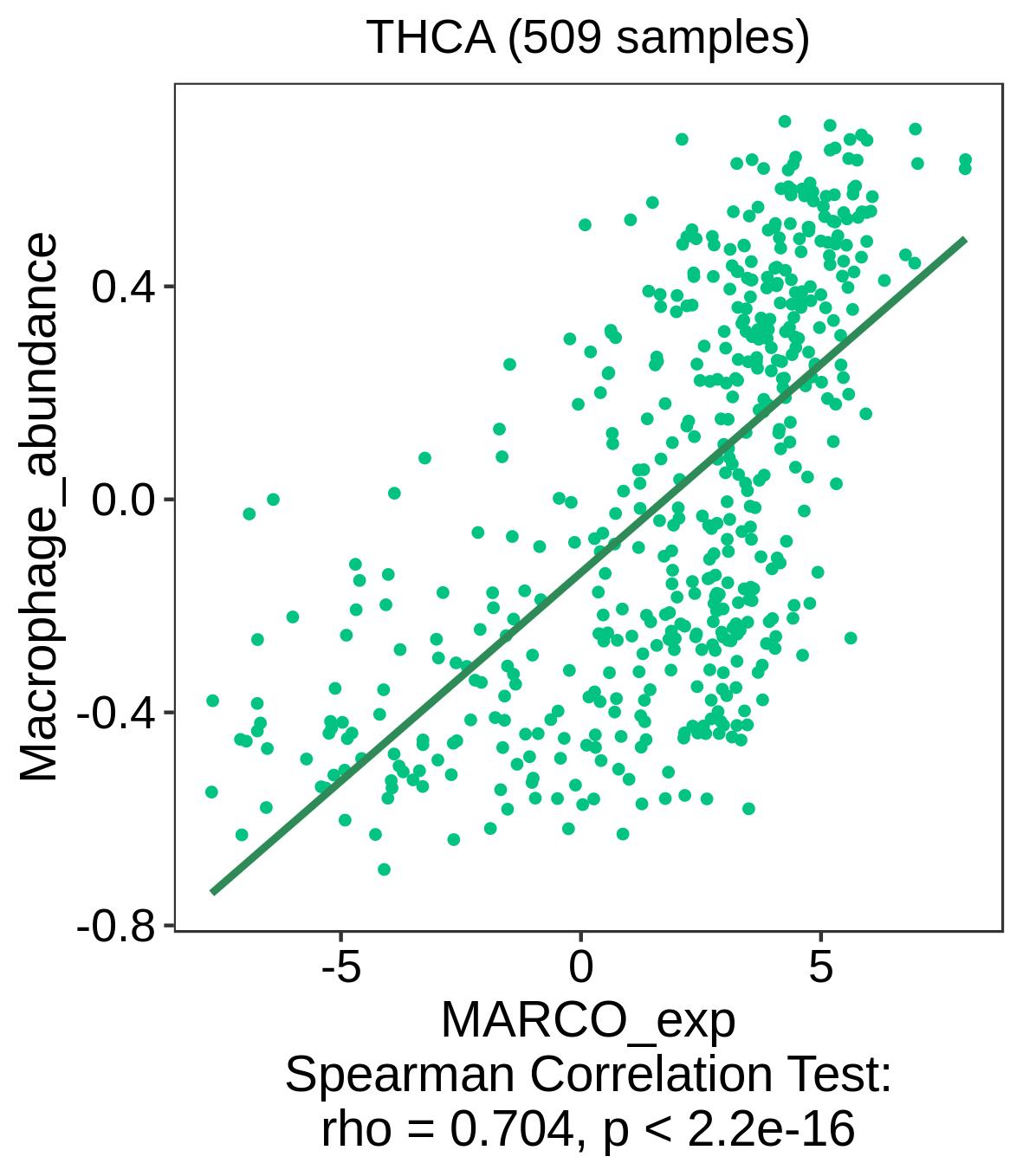


**Supplementary Figure 9.** The association between MARCO expression and macrophage infiltration in THCA.


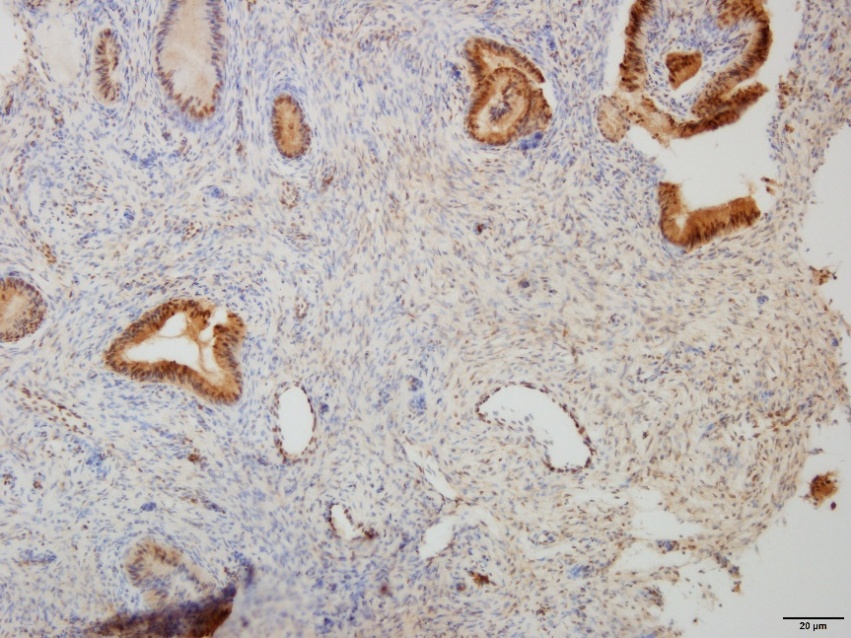


**Supplementary Figure 10.** Expression of CD68 in cervical tissues of patients with HPV infection, scale bars = 20 μm.


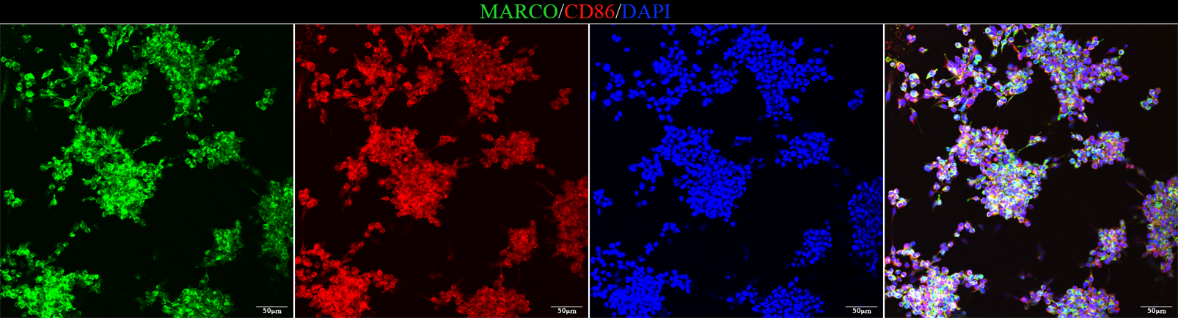


**Supplementary Figure 11.** Fluorescence Double Staining of MARCO and CD86 in Macrophages after Nr-CWS Treatment, scale bars = 50 μm.


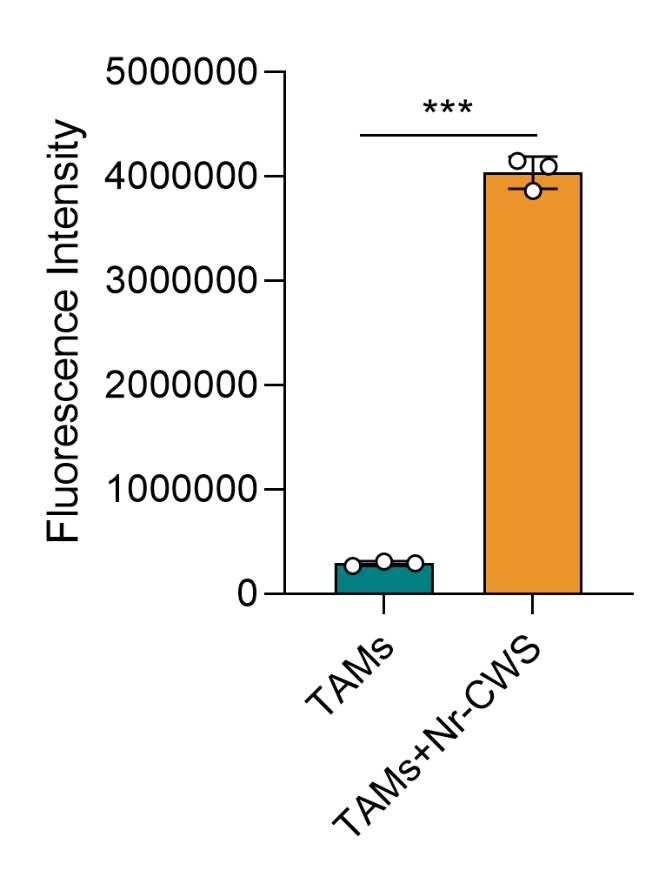


**Supplementary Figure 12.** The phagocytosis of HeLa cell debris by macrophages before and after Nr-CWS treatment. n = 3, ^***^*P* < 0.001.


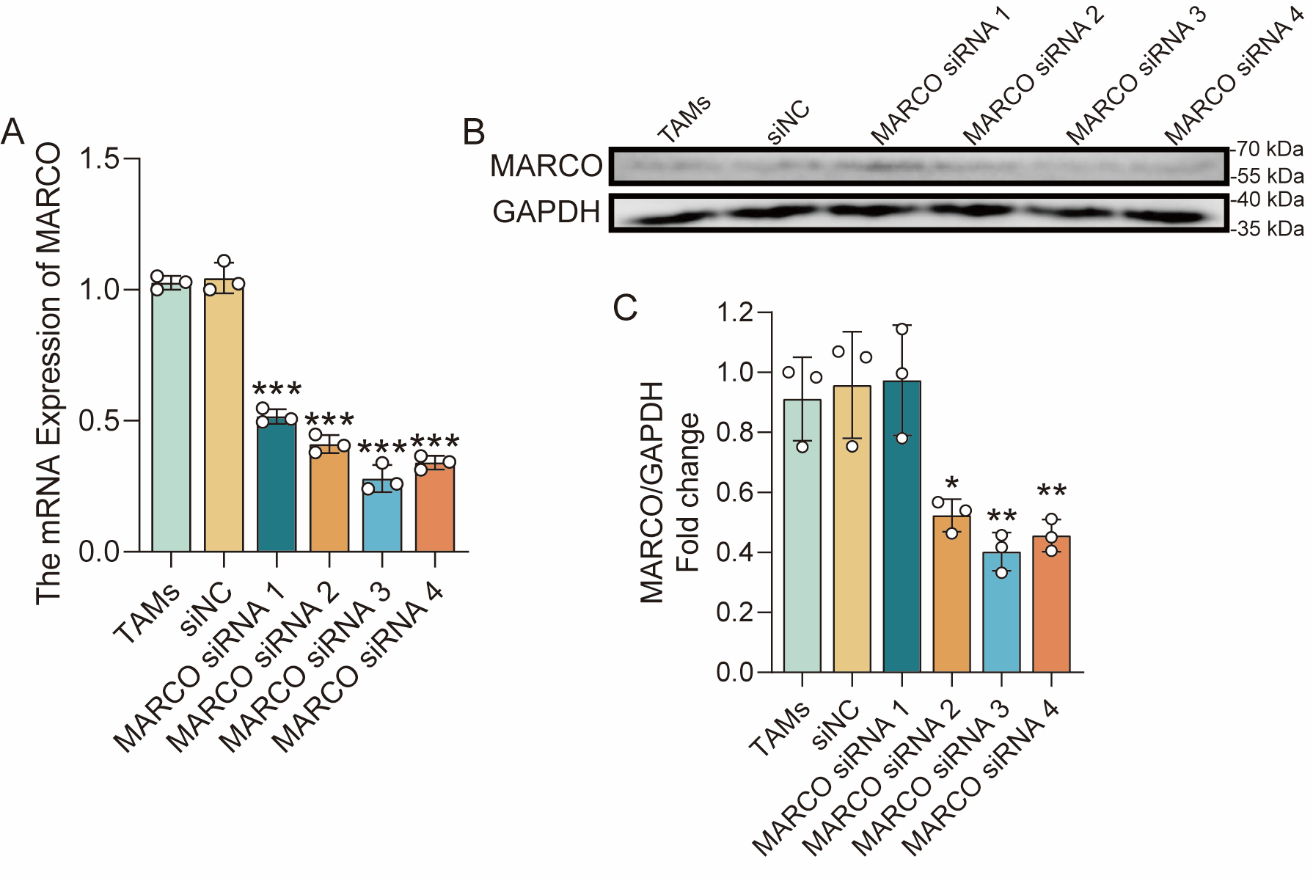


**Supplementary Figure 13.** Interference effect of small interfering RNA. The expression level of MARCO mRNA detected by RT-qPCR (A) and WB (B and C). n = 3, ^*^*P* < 0.05, ^**^*P* < 0.01, ^***^*P* < 0.001 vs. siNC.
